# Isotope Effects on the Vaporization of Organic Compounds from an Aqueous Solution–Insight from Experiment and Computations

**DOI:** 10.1021/acs.jpcb.1c05574

**Published:** 2021-12-15

**Authors:** Michał Rostkowski, Heide K. V. Schürner, Agata Sowińska, Luis Vasquez, Martyna Przydacz, Martin Elsner, Agnieszka Dybala-Defratyka

**Affiliations:** † Institute of Applied Radiation Chemistry, Faculty of Chemistry, Lodz University of Technology, Zeromskiego 116, 90-924 Lodz, Poland; ‡ Chair of Analytical Chemistry and Water Chemistry, Technical University of Munich, Elisabeth-Winterhalter-Weg 6, 81377 Munich, Germany

## Abstract

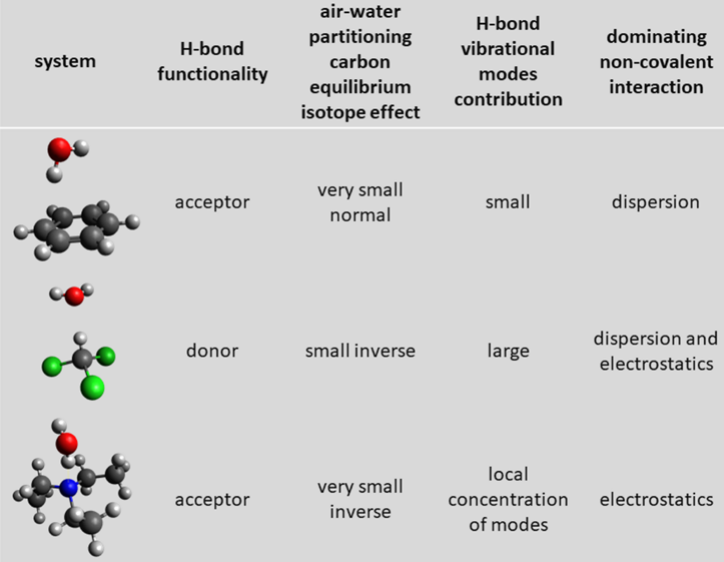

An isotope fractionation analysis of organic groundwater pollutants can assess the remediation at contaminated sites yet needs to consider physical processes as potentially confounding factors. This study explores the predictability of water–air partitioning isotope effects from experiments and computational predictions for benzene and trimethylamine (both H-bond acceptors) as well as chloroform (H-bond donor). A small, but significant, isotope fractionation of different direction and magnitude was measured with ε = −0.12‰ ± 0.07‰ (benzene), ε_C_ = 0.49‰ ± 0.23‰ (triethylamine), and ε_H_ = 1.79‰ ± 0.54‰ (chloroform) demonstrating that effects do not correlate with expected hydrogen-bond functionalities. Computations revealed that the overall isotope effect arises from contributions of different nature and extent: a weakening of intramolecular vibrations in the condensed phase plus additional vibrational modes from a complexation with surrounding water molecules. Subtle changes in benzene contrast with a stronger coupling between intra- and intermolecular modes in the chloroform–water system and a very local vibrational response with few atoms involved in a specific mode of triethylamine. An energy decomposition analysis revealed that each system was affected differently by electrostatics and dispersion, where dispersion was dominant for benzene and electrostatics dominated for chloroform and triethylamine. Interestingly, overall stabilization patterns in all studied systems originated from contributions of dispersion rather than other energy terms.

## 1 Introduction

Monitoring the transformation of organic pollutants in the environment is important, especially in groundwater systems where chemical pollution needs to be evaluated at contaminated sites.^1−3^ Often, however, degradation is difficult to assess based on mass balances. An attractive alternative is to utilize changes in the isotopic composition of pollutants caused by kinetic isotope effects when the molecules undergo a chemical transformation.^4−6^ The isotopic composition of entire compounds can be studied with a compound-specific isotope analysis (CSIA) that measures stable isotope ratios for a given element at natural abundance and can be used to determine the difference in the isotope composition between different samples.^7^ Since the magnitude of the isotopic fractionation is related to the process these compounds undergo, that is, chemical, biochemical, or physical change, this approach allows one to trace the pathways by which contaminants or other compounds of interest spread and are decomposed.^8,9^

**Scheme 1:**
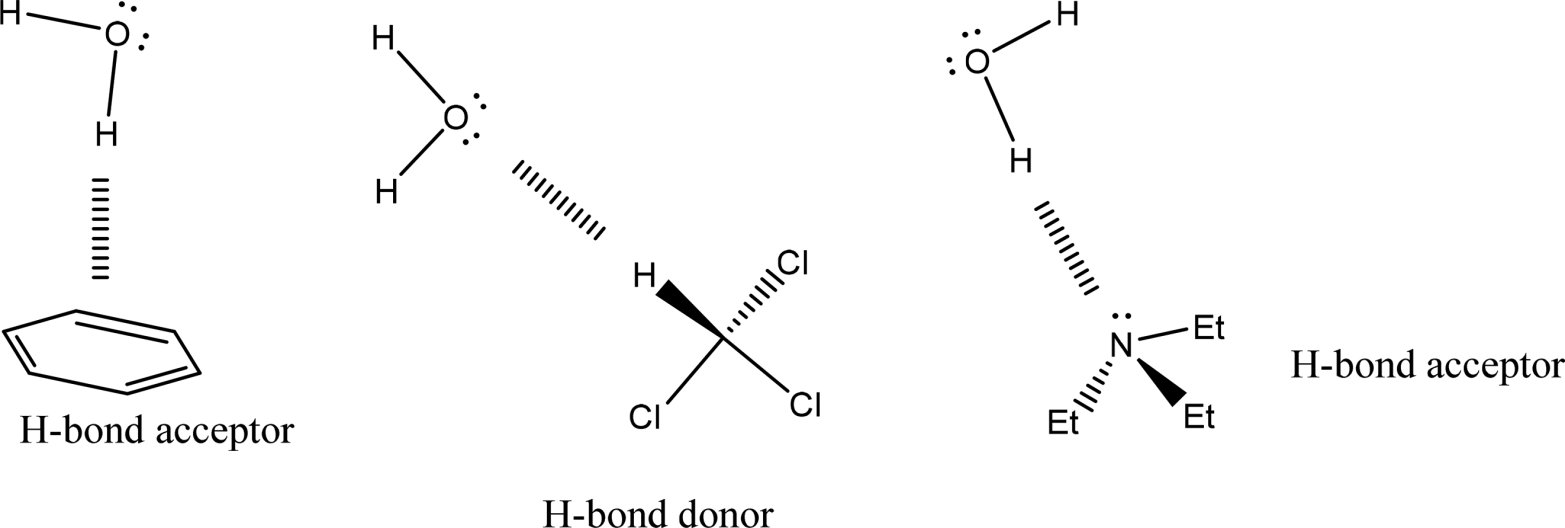


The approximation is frequently made that only (bio)chemical transformation processes alter the isotopic composition of contaminants significantly and that other processes such as dilution, evaporation, and sorption–desorption play a minor role. This assumption has been repeatedly challenged,^10−14^ so it is important to understand the direction and magnitude of isotope effects on partitioning to interpret degradation-associated isotope fractionation correctly. In particular, evaporation—an important pathway of volatile organic compound attenuation—may potentially cause non-negligible changes in the isotopic composition of these organic compounds. When a heavier isotopologue accumulates in the liquid phase, the observed effect is called a normal isotope effect. In contrast, when an initial (liquid) state becomes enriched in lighter isotopologues, meaning that heavier moieties are more prone to evaporation, then we call such an effect inverse. Previous studies have substantiated the general rule that an observable kinetic isotope fractionation of a liquid phase-air transfer will reflect the bottleneck of the overall process.^10,15−21^ If diffusion through the liquid phase is rate-limiting, the observable isotope effect that reflects a liquid phase diffusion will typically be very small.^19,22^ If, however, diffusion through a stagnant air layer above the water surface is rate-limiting, the overall isotope effect may be non-negligible, and it will be a composite of the equilibrium air/solvent isotope effect and the kinetic isotope effect of diffusion through air. While isotope effects of diffusion in the gas phase can be estimated based on isotopologue masses (see, e.g., Bouchard 2011^16^), the equilibrium isotope effect of solvent-air partitioning is less straightforward to predict. Since such partitioning isotope effects arise from changes in internal motions of a compound that are caused by specific interactions with surrounding solvent molecules, one may expect a correlation with the nature of these noncovalent interactions. To probe this hypothesis, we approached the problem from two directions. First, in an experimental approach we investigated equilibrium isotope effects in water–air partitioning for (i) benzene as a model compound for a π-electron system able to accept hydrogen bonds, (ii) trichloromethane as a likely hydrogen-bond donor, and (iii) trimethylamine as a hydrogen-bond acceptor (see Scheme 1). Second, in the computational approach we first aimed to reproduce the experimental results by exploring several combinations of theory levels and solvation models. Subsequently, we used successful combinations to interpret them in terms of underlying intermolecular interactions (i.e., changes in intermolecular vs intramolecular vibrational modes) to assess whether a dependency between intermolecular interactions and isotope effects can indeed be expected.

### Hypotheses and Experimental Design

To investigate the effect of specific intermolecular interactions, the design of our experimental study targets the partitioning between air and water as the most important natural solvent. Every substance is affected by interactions between various dipoles, permanent and induced ones (often called van der Waals forces) when it condenses from the gaseous to the liquid phase, due to the dispersive forces between polarizable molecules leading to attraction. In contrast, hydrogen bonds are additional attractive interactions that can only form with elements of the second period of the periodic table when a hydrogen atom that is covalently bound to a highly electronegative atom (e.g., oxygen, nitrogen, or fluorine) acts as donor and a lone electron pair acts as hydrogen-bond acceptor.

The equilibrium constant *K*_*i*_ for the partitioning of compound *i* between two phases is given as  with *c*_*i*_ as the concentration in the respective phase. Correspondingly, equilibrium isotope effects (EIEs) are expressed as
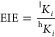
1with superscripts “l” and “h” indicating light and heavy isotopologues, respectively. An isotope effect on the equilibrium constant can be obtained from a ratio of isotopic partition functions, which is a result of different molecular vibrations for heavy and light isotopes, respectively.^23^ In the gas phase only *intra*molecular vibrations (i.e., within covalent bonds) occur. Their contribution to vapor pressure equilibrium isotope effects is represented by term *B* of eq 2, where *P*^l^ and *P*^h^ are the vapor pressures of light and heavy isotopologues, respectively.
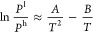
2eq 2 can be linked to the EIE by^24^
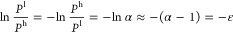
3and
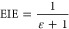
4By a condensation of a molecule from the gas phase to the liquid phase, these *intra*molecular vibrations (term *B*) are weakened, but at the same time, new additional vibrational modes are created that did not exist before (i.e., *inter*molecular interactions, e.g., induced by van der Waals forces or hydrogen bonds). The contribution of these *inter*molecular interactions is represented by term *A* in eq 2. At low temperatures, term *A* dominates so that the isotope effect of volatilization is always normal. In contrast, term *B* becomes more and more important with increasing temperatures leading to a temperature at which the isotope effect may change from normal to inverse. At very high temperatures, finally, both terms become zero, and isotope effects are nonexistent. For more details on eq 2 see the review of Jancso and Van Hook^25^ on condensed-phase isotope effects. When considering that the vibration of hydrogen bonds is of a higher energy than that of van der Waals forces, it is expected that *inter*molecular vibrations of hydrogen bonding give greater contributions to term *A* than van der Waals interactions, meaning that this term could dominate a larger temperature range in the case of hydrogen bonds. Hence, on the one hand, compounds interacting at room temperature via van der Waals interactions may show an inverse isotope effect when they partition from a condensed phase into the gas phase because term *B* of eq 2 is already dominating at this temperature. For compounds interacting at room temperature via hydrogen bonding, on the other hand, term *A* could still dominate, and these substances are expected to show a normal isotope effect. Evidence for this is, for example, given by Zhang et al.^26^ who conducted a Rayleigh distillation of pure compounds including alcohols and observed normal hydrogen and oxygen isotope effects for the −OH groups (where hydrogen bonding was important) and inverse carbon and hydrogen isotope effects for the alkyl chains (where van der Waals interactions dominated).

### Computational Approach

Theoretical models provide a powerful tool for interpreting experimental findings by enabling direct insight into the influence of the solvent–solute interactions on differences in the partitioning of different isotopologues between air and water at the atomic level. Since isotope effects discussed in this work are very small, a reliable theoretical description of model systems as well as an adequate model of the phenomena under study to reproduce such small isotopic fractionation is warranted. One of the simplest and most natural approaches for modeling the process of interest is having a liquid phase (water) represented by a proper solvation model and a gas phase (air) by only one molecule in vacuo. The problem of a proper theoretical description of solvated systems is twofold. First, an appropriate theory level must be chosen for calculations, and second, solvent representation is also a crucial choice. To perform meaningful calculations, it is therefore necessary to find a compromise between the model size and an accurate level of a theory to describe nuclear motions of a system correctly, especially due to the necessity of reproducing weak interactions between polar solvent and organic molecules studied herein.^27−30^ This usually requires one to apply quantum-chemical calculations at least to the solute molecule if not to the directly interacting solvent molecules as well. Such a strategy however severely limits the model size one can consider. One way of dealing with such limitations for bigger models is so-called mixed or hybrid cluster-continuum solvent models. Another approach will comprise the full explicit presence of solvent molecules in a large number, allowing one to capture important short-range interactions as well as bulk solvation effects.

## 2 Methodology

### Chemicals

Benzene was purchased from Merck (Darmstadt, Germany), and triethylamine (TEA), trichloromethane (TCM), and deuterotrichloromethane (CDCl_3_, 99.96 atom % D) were from Sigma-Aldrich (Steinheim, Germany). All organic substances were of liquid chromatography-mass spectrometry (LC-MS) grade (purity >99%). Milli-Q water was generated by a Milli-Q Advantage A10_system_ from Millipore (Schwalbach, Germany).

### Experimental Setup

Water stock solutions (∼1 g/L benzene, ∼6 g/L TEA, and ∼5 g/L TCM) were prepared in concentrations within the water solubility range of these compounds. To avoid air bubbles, 250 mL glass bottles were filled completely with water and closed with Mininert screw caps before the substances were injected by a syringe. Afterward, the solutions were stirred rigorously for 48 h.

**Figure 1:**
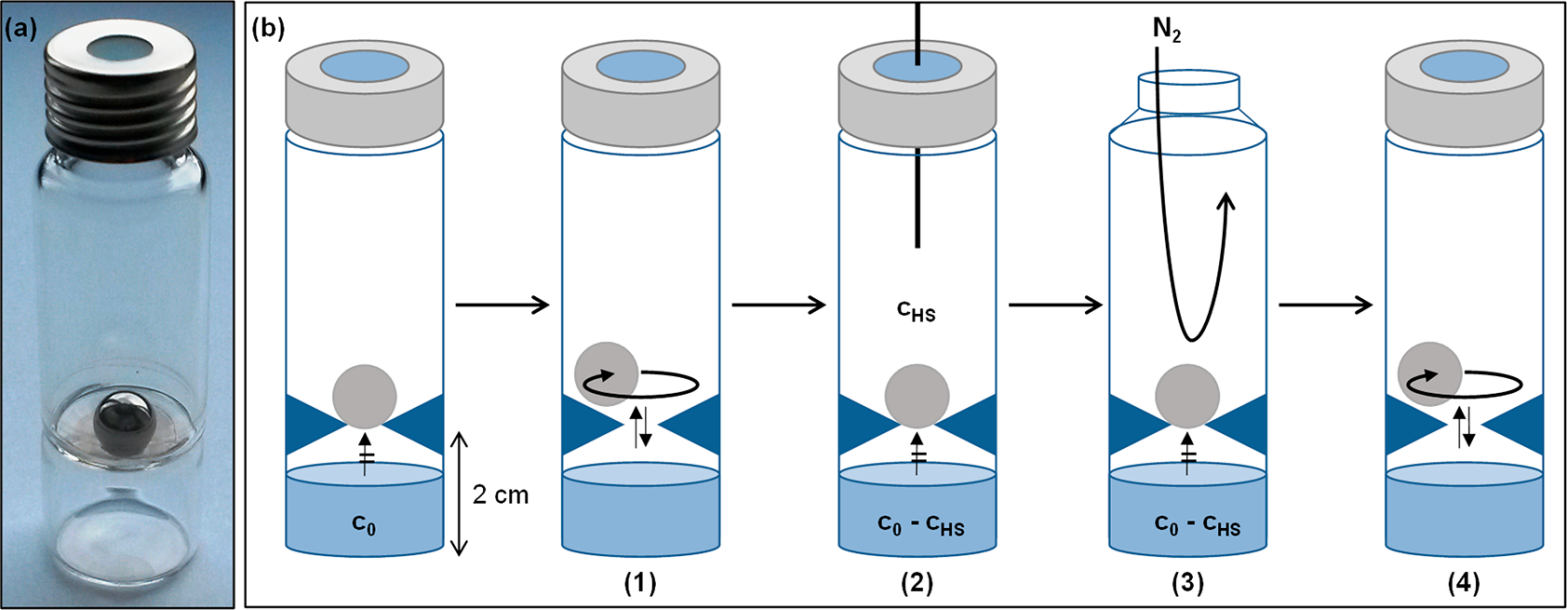
(a) Modified headspace vial. (b) Schematic illustration of the stepwise water–air partitioning: (1) equilibration in agitator; (2) automated headspace sampling; (3) exchange of headspace with gentle nitrogen stream; (4) repeat (1–3).

To avoid bias from isotope effects of diffusion^19^ water–air partitioning experiments were performed with a stepwise equilibration in modified ND18 headspace vials (Figure 1). The vials were divided into two parts by a tapered grinding (2 cm from the bottom) resulting in an upper vial volume of ∼14 mL and a lower vial volume of ∼4 mL, with a small hole to which a ball (stainless steel, diameter: 0.9 cm) was fitted. The lower part of the vial was filled with a stock solution (Figure 1), the ball was added, and the system was closed with a screw cap (1.3 mm silicone/polytetrafluoroethylene (PTFE) septum, 45°, Carl Roth, Karlsruhe, Germany). The amount of stock solution used was calculated to achieve a 50:50 distribution by mass between the headspace and water. The distribution (in %) between two equilibration steps *n* can be calculated according to
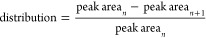
5

After equilibration in the autosampler agitator (speed: 500 rpm, incubation temperature: 33 °C, equilibration time: see Table S1) the ball closed the hole, and headspace samples were taken automatically. Afterward, the vial was opened manually, the gas phase was exchanged with a gentle nitrogen stream for 20 s, and the vial was quickly closed with a new screw cap. The substance loss during the manual gas-phase exchange was determined to be less than ∼1.4% of the total amount after 30 s (data not shown). Experiments were performed in duplicates. For quantification, standards prepared from stock solutions were added to unmodified ND18 headspace vials (with the same volume that was used for the experiments).

### Compound-Specific Stable Isotope Analysis (CSIA) of Water–Air Partitioning

A carbon isotope analysis of benzene, triethylamine, and trichloromethane was conducted on a gas chromatography infrared mass spectrometry (GC-IRMS) system using a TRACE GC Ultra gas chromatograph (Thermo Fisher Scientific, Milan, Italy), which was coupled to a Finnigan MAT 253 isotope ratio mass spectrometer via a Finnigan GC Combustion III interface (both from Thermo Fisher Scientific, Bremen, Germany). For benzene, an Rxi-5Sil MS analytical column (30 m, 0.25 mm ID, 1.0 μm film) from Restek (Bad Homburg, Germany) was used. The initial GC oven temperature was 50 °C (hold 3 min), ramped at 40 °C/min to 120 °C (hold 2 min), and ramped at 55 °C/min to 280 °C (hold 5 min). The total uncertainty of benzene measurements was ±0.2‰. Triethylamine and trichloromethane were analyzed using a DB-624 analytical column (60 m, 0.25 mm ID, 1.4 μm film, Agilent Technologies, Böblingen, Germany). The GC oven program started at 120 °C (hold 12 min) and was ramped at 100 °C/min to 280 °C (hold 2 min). The standard deviation of triethylamine and trichloromethane was ±0.4 and ±1‰, respectively.

δ^13^C values are expressed in per mil relative to Vienna PeeDee Belemnite (V-PDB).
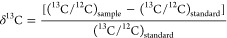
6A hydrogen isotope analysis of a trichloromethane and deuterotrichloromethane mixture (1:1, v/v) was performed on a GC-MS system in selected ion monitoring (SIM) mode. An Agilent 7890A GC system (Agilent Technologies, Santa Clara, United States) equipped with a DB-5 analytical column (30 m, 0.25 mm ID, 1.0 μm film, Agilent Technologies, Böblingen, Germany) was coupled to an Agilent 5975C mass-selective triple-axis detector (Agilent Technologies, Santa Clara, United States). The initial GC oven temperature was 120 °C (hold 5 min), ramped at 50 °C/min to 280 °C (hold 6 min).

^2^H/^1^H ratios are expressed in per mil using the following equation.
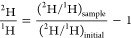
7

**Figure 2:**
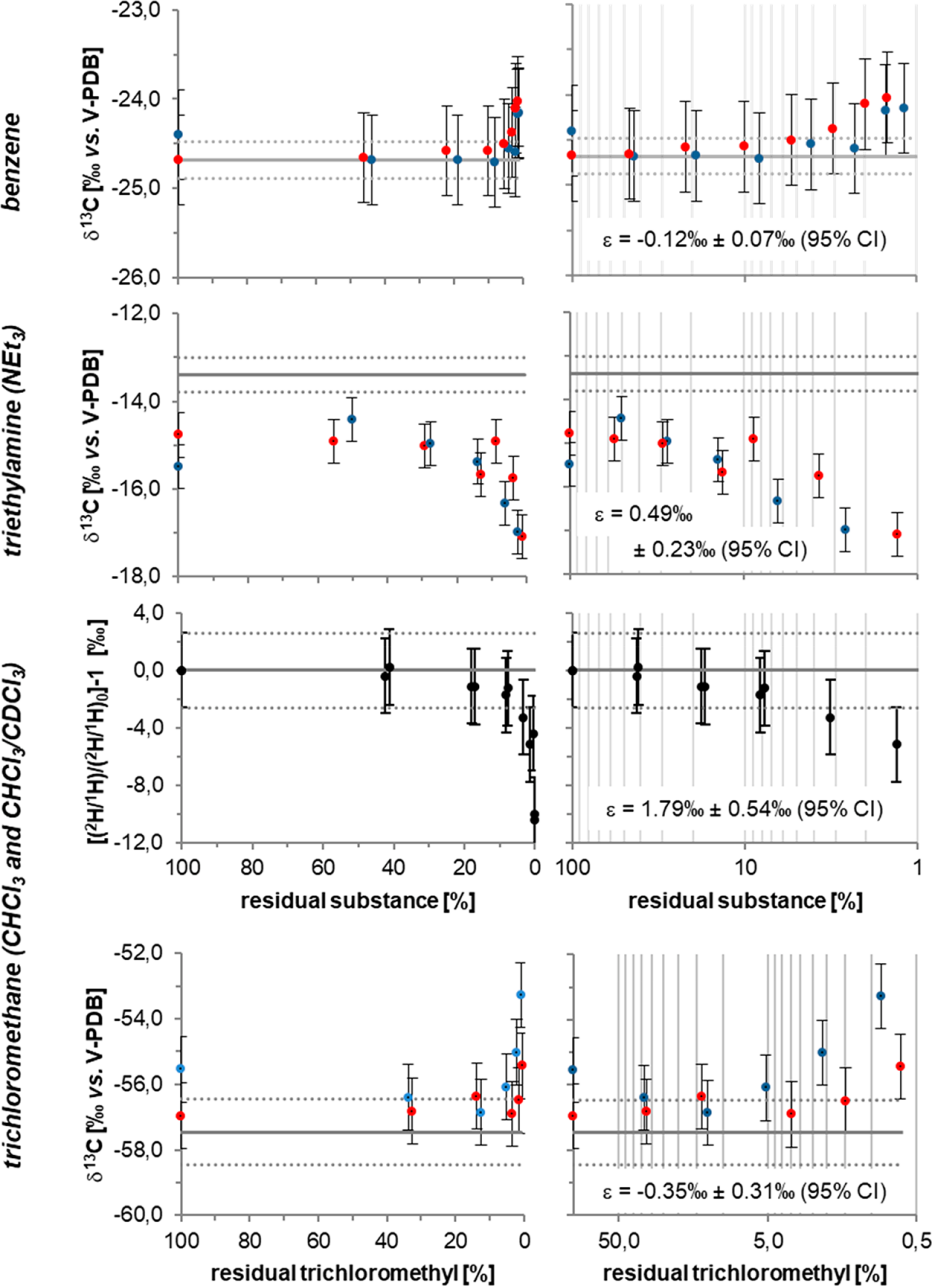
Carbon isotope data of benzene, triethylamine, and trichloromethane as well as hydrogen isotope data of trichloromethane determined from a CHCl_3_/CDCl_3_ mixture measured during water–air partitioning. For a carbon isotope analysis, gray lines represent the isotopic composition of the standard determined by EA-IRMS. For hydrogen isotope analysis, the gray line denotes the measured initial average. Dotted lines and error bars represent the precision of carbon (±0.5‰ for benzene and TEA (NEt_3_), ±1‰ for TCM) and hydrogen (±2.6‰) isotope analyses. Reported enrichment factors ε with 95% CI were obtained according to the Rayleigh equation (eq 8) by a linear regression of isotope values vs the logarithm of concentrations as represented in the panels to the right.

A calculation of the enrichment factor ε for stepwise equilibrium exchange reactions is possible using (i) an approach considering consecutive equilibrium steps or (ii) the classical Rayleigh equation considering a continuous process. A comparison in the Supporting Information (Table S2) gives essentially the same results for both evaluation methods so that—consistent with earlier findings^31^ by Jeannottat and Hunkeler—the Rayleigh eq (eq 8) was used in this study according to
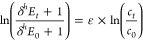
8where the isotopic signatures of element E are given as δ^h^E_*t*_ (at time *t*) and δ^h^E_0_ (at the beginning of the experiment), and *c*_t_/*c*_0_ is the fraction of the remaining substrate after a stepwise exchange of the headspace in a partitioning experiment corresponding to data points in Figure 2. In the case of normal isotope effects (i.e., molecules containing the light isotopologues escape preferentially into the gas phase), ε-values are negative, while positive ε-values indicate inverse isotope effects.

### Computational Approach

The presence of a solvent can be introduced in computational studies in two basic ways that differ not only in the general concept but also in the amount of information they can provide on the modeled system. The simplest solvent description is an implicit or so-called continuum solvent model that introduces the solvent mathematically as a polarizable continuum (field).^32^ Such calculations are fairly quick and feasible to perform, in general, even for bigger molecular systems. In the present work, this implicit, structureless representation of the solvent was included by applying a polarizable continuum solvation model (PCM).^33^ As this model lacks noncovalent interactions characteristic of the studied system, for example, hydrogen bonds, to account for specific solute–solvent interactions, originating from the presence of the first solvation shell of solvent molecules, explicit water molecules were either placed manually in the near vicinity of a solute molecule or were cut out from the prepared water boxes within a given distance from a centrally located solute molecule. This procedure resulted in cluster models varying in size (number of water molecules included within). In the former case, water molecules were located manually in proximity to a solute molecule and oriented in a way to reproduce possibly the most likely specific interactions between the solvent and a solute. For the latter approach, coordinates of water boxes were prepared with the tLEAP program, available as one of the AmberTools in the Amber18 package.^34^ In general, each solute molecule was immersed in the TIP3P^35^ solvent water box. Resulting geometries were processed in VMD 1.9.3,^36^ where closest surrounding water molecules were selected based on their distance to the solvated molecule. Structures were further used as input geometries for electronic structure calculations, both with and without additional continuum solvation models—representing mixed and microsolvation models, respectively. For these calculations we limited ourselves to use the three following density functional theory (DFT) functionals: B2PLYP with Grimme’s D3 dispersion correction^37^ (B2PLYP-D3^38,39^), M06-2X,^40^ and B3LYP.^41,42^ Dispersion corrections were shown to improve the performance of the DFT, especially in the case of polar water interactions with aromatic systems.^43^ Besides, in one of the latest and most extensive benchmarks^44^ on DFT chemistries, B2PLYP-D3 performed best among the other benchmarked functionals for noncovalent interactions, having a weighted total mean absolute deviation (WTMAD) equal to 3.8 kcal·mol^–1^. Moreover, this is the only functional in that study that contained an explicit dispersion correction. A benchmark showed that, for the Minnesota-type functional M06-2X, a WTMAD value as low as 6 kcal·mol^–1^ can be expected. It is worth mentioning that, specifically for this functional, little or no accuracy gain is seen from the introduction of dispersion corrections. Finally, although the B3LYP functional without dispersion corrections generates a WTMAD of 25.72 kcal·mol^–1^ for noncovalent interactions, in the past B3LYP performed reasonably well for other studies, perhaps due to error cancellations, and is still one of the more popular functionals; we decided to include it for comparison. These DFT functionals were combined with two split valence basis sets, 6-31+G(d,p) and 6-311+(2df,2p),^45^ referred to in the text as the smaller and the larger basis set, respectively.

All initial cluster model structures were optimized to locate nearest minima on the potential energy surface prior to the normal-mode analysis. Larger solvation cluster models for the systems prepared manually were also additionally preoptimized with the PM3^46^ semiempirical method. Electronic structure calculations were performed with the Gaussian 09 ver. D01 program^47^ using the tight convergence criteria for optimizing structures of stationary points. Formatted checkpoint files were processed in the TAMkin program^48^ using in-house scripts to perform an isotopic substitution in mass-weighted Hessian matrices, which were subsequently subjected to equilibrium isotope effect calculations based on the Bigeleisen equation (eq 9),^49^ by calculating the ratios of respective frequencies for different isotopologues.
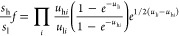
9Here, *u*_*i*_ = *hcv*_*i*_/*kT*, and *v*_*i*_ is the *i*th vibrational mode of the lighter (l) and heavier (h) isotopologues, respectively.

Solvated systems were considered as an initial (reactant) state in a vaporization process, whereas a solute molecule alone was treated in vacuo as a final (product) state of this process. The isotopic fractionation of carbon obtained that way was subsequently averaged over all carbon atomic positions to compare the predicted isotope effect (IE) value to the corresponding experimental data. Computed isotope effects are presented as isotopic enrichment factors, expressed in ‰, which are related to equilibrium isotope effects via eq 4.

Large models consisting of an explicit representation of all solvent molecules were also constructed. For this purpose, initial coordinates of each solute were taken from the respective gas-phase calculations at a DFT level of theory. Next, each studied compound was solvated in a box of water molecules using the TIP3P water model using the tLEAP module of the Amber18 package. The resulting systems consisted of 9300, 8900, and 10 195 atoms for benzene, chloroform, and triethylamine, respectively. Each system was subjected to the standard sequence of minimization-heating-equilibration-production calculation/simulation as described in detail in the Supporting Information. Selected structures for each system were optimized using the quantum mechanics/molecular mechanics (QM/MM) protocol within which the QM region comprised the organic molecules and the MM part consisted of all water molecules treated by TIP3P. The QM method was selected based on the tests performed for the cluster models discussed in this work. Details of this part of the calculations can be also found in the Supporting Information.

Water–air partitioning EIEs were predicted without scaling the vibrational frequencies, at the DFT/MM using the Bigeleisen equation as described above for the cluster models. However, the TAMkin software was not used for this purpose.

In IE calculations atoms from the QM region (the solute atoms) were used to define the Hessian by considering the “cut-off rule” and the local nature of isotope effects. IEs were computed by applying rigid-rotor and harmonic oscillator approximations. In this work, these partial Hessians that resulted from the QM/MM treatment of the systems were subjected to a standard projection procedure to remove translational and rotational functions, giving rise to 3N_s_-6 frequencies, where N_s_ is a subset of atoms treated quantum mechanically and subjected to the Hessians calculations in the presence of the environment. Additionally, no projection was applied, which resulted in the 3N_s_ set of vibrational frequencies. In this way, we could assess whether these six librational modes contribute to the predicted isotope effects.^50^ The combination of 10 (TCM) or 60 (benzene and TEA) IE individual values obtained with 10 solute–solvent structures and one gas-phase structure allowed for the calculations of bulk EIEs with their uncertainties (see Table S7a,b).

For TCM, 10 individual values were obtained. For benzene and TEA, as they possess six carbon atoms, 10 values for each carbon position within a molecule were calculated, which summed up to 60 values of carbon isotope effect per compound (either benzene or TEA). They were average values as well as standard deviations, and standard errors were calculated.

The intermolecular interaction energy Δ*E*^inte^ was computed between the noncovalent complex of monomers (the solvent and solute molecule) as well as for pairs of separate solvent molecules interacting with solute.^51^ Thus, for a complex XY containing monomers X and Y, its interaction energy is expressed as follows
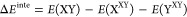
10where X and Y superscripted with XY mean that the energy of each monomer is computed in the basis set of the whole complex. Calculation of monomer energies in the complex basis set allows one to consider a presence of the remaining part of complex instead of calculating their energies separately. This treatment is known as a counterpoise correction^52^ and is used to avoid the so-called basis set superposition error (BSSE).^53,54^

Within the symmetry-adapted perturbation theory (SAPT),^55^ intermolecular interaction energy is given as a perturbation series that can be assigned to four fundamental physical components, namely, electrostatics *E*_elect_, induction *E*_ind_, dispersion *E*_disp_, and exchange-repulsion *E*_exc_.
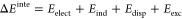
11Electrostatics describes Coulomb interactions between permanent charge densities of interacting monomers.^56^ Induction (polarization) can be interpreted as the response of the electron density of one monomer to the electric field of the permanent charge distribution of the other monomer, that is, mutual polarization between the two molecules. The dispersion is when both interacting monomers are polarized. It results from the quantum correlation of the electronic motions between the monomers and does not have a classical interpretation. The exchange energy term is sometimes called the valence-repulsion energy, since it is proportional to the overlap of monomer’s wave functions. It can be interpreted as the effects of the electron tunneling through the potential barrier between the interacting monomers.

The higher the order at which calculations are realized, the more interaction perturbation terms are computed; thus, a more accurate interaction energy can be obtained. SAPT calculations^57^ were performed with the PSI4 program,^58^ ver. 1.2.1, using the correlation-consistent augmented triple Dunning basis set (aug-cc-pVTZ^59,60^) supermolecular MP2 interaction energy (δMP2), and coupled-cluster doubles (CCD) for dispersion^61^ on the optimized geometries of the studied cluster structures in order to describe the studied intermolecular interactions at a reasonable level.^62,63^ For the sake of convenience, the chosen method can be abbreviated as SAPT2+(CCD)δMP2/aTZ, but SAPT will be used here instead, as it was the only SAPT method used in this work. Since SAPT was developed mainly for two-body interactions, the interaction energy for clusters containing more than one water molecule was calculated as a total sum of interaction energies between the solute and each of the surrounding solvent molecules present in a given cluster separately, as a solvent–solute dimer. Besides the absolute interaction energy obtained from the SAPT calculations, a ratio of the dispersion forces to the electrostatics (*D*/*E*)^64^ as well as ratios of other intermolecular interaction terms to the electrostatics were computed in order to highlight the relative contribution of each type of interaction.

## 3 Results

### Experimentally Determined Air–Water Partitioning Isotope Effects

Determining very small isotope effects with sufficient precision is experimentally challenging. Previous studies have measured isotopic differences between different phases in one-step partitioning experiments. Here we aimed for a magnification of the observable isotope fractionation by conducting partitioning experiments in multiple steps similar to those of Jeannottat and Hunkeler,^31^ where each step aimed for a 50:50 distribution between the headspace and water, and where after each step the headspace was sampled and subsequently removed. As can be seen in Figure S1, the desired 50:50 distribution of the substances could approximately be achieved during the water–air partitioning. From the data presented in Figure S1 the experimental setup used in this study (Figure 1) appears, therefore, promising to determine isotope fractionation in partitioning experiments.

As shown in Figure 2, during stepwise water–air partitioning ^13^C became enriched in the benzene of the aqueous phase corresponding to a normal isotope effect of volatilization (i.e., light isotopologues escaped preferentially) with ε = −0.12‰ ± 0.07‰ (95% confidence interval (CI)). In comparison, the tertiary amine triethylamine, which provides a hydrogen bond acceptor moiety at the nitrogen atom, showed a significant inverse carbon isotope effect of ε = 0.49‰ ± 0.23‰ (95% CI). Finally, carbon isotope values of trichloromethane showed practically no significant trend (ε = −0.35‰ ± 0.31‰, 95% CI).

Figure 2 seems to indicate a discrepancy between (a) the ε value obtained from the difference between the headspace and the original substance (EA-IRMS characterization, solid lines in Figure 2) and (b) the ε value obtained from analyses of the residual substance in an aqueous solution sampled from the headspace after multiple equilibration steps (trend in Figure 2 evaluated according to eq 8). If the TEA in the headspace of the first equilibration step (100% residual substance) really contained less ^13^C than the original substrate, this would imply a normal isotope effect, meaning that residual TEA should become increasingly enriched rather than depleted in ^13^C during the experiment—in contrast to our observations in Figure 2. In response we note that our experimental setup was not optimized to ensure absolute isotopic traceability of the starting value, as also indicated by the differences between replicates (red and blue data points in Figure 2). Hence, to rely on the difference between headspace and aqueous solution, we would have had to aim for an exhaustive alternating analysis of both phases from the same vial. We, therefore, refrain from such interpretations of our experiments and report EA-IRMS values simply for transparency and to illustrate the general challenges associated with precise determinations of isotope effects at such a small magnitude.

Further, we note that the negligible carbon isotope effect obtained for chloroform is in contradiction with the inverse isotope effect of ε = 1.5‰ ± 0.3‰ (95% CI) reported by Hunkeler et al.^65^ and of ε = 1.4‰ ± 0.2‰ (standard error) recently reported by Horst and Lacrampe-Coloume.^21^ This discrepancy may exemplify the challenge of determining isotope effects of such a small magnitude and to reconcile different experimental approaches. Whereas the other studies analyzed chloroform in the headspace over an aqueous solution in a one-step experiment, we derived equilibrium isotope fractionation from analyses of the residual aqueous chloroform after multiple equilibration steps. While we feel that our approach is ideally suited to eliminate any measurement bias, because effects would magnify by the repeated extraction procedure, we acknowledge that the results of the other two studies are in perfect agreement. In the following they are, therefore, used as a benchmark for our computations.

In the case of hydrogen a significant inverse isotope effect was observed for volatilization of the CHCl_3_/CDCl_3_ mixture from an aqueous solution (ε = 1.79‰ ± 0.54‰, 95% CI, Figure 2), which is in qualitative agreement with recent data from Horst and Lacrampe-Coloume,^21^ who determined an even greater inverse isotope effect of ε = 7.4‰ ± 2.7‰ (standard error).

### Computed Water–Air Partitioning Isotope Effects at Different Levels of Theory

IEs presented herein were obtained in quantum-chemical calculations using different solvent representations, beginning with the least computationally expensive continuum model via the cluster models in which explicit solvent–solute interactions were present in a varying number and were all treated at the same quantum level of theory to full explicit solvent models in which the solute atoms treated quantum mechanically were surrounded by a large number of solvent molecules described using an empirical force field. The outcome was referenced to the experimentally evaluated isotope fractionation of the three representative organic compounds, that is, benzene, trichloromethane, and triethylamine. Missing values in the tables are a result of convergence problems in locating stationary points by optimization algorithms for the studied many degrees-of-freedom systems. The results obtained for large pure explicit models due to the specificity of calculations are presented separately.

#### 3.1 Continuum Solvation Models

First, the solvation of benzene, trichloromethane, and triethylamine was represented only by the PCM of water. Isotope effect values obtained based on calculations performed at various theory levels are collected in Tables S3–S5. In the case of benzene all obtained ε values were positive, within a range from 0 to ∼0.5‰, which indicated a small inverse isotope effect, not corresponding to the experimental normal isotopic fractionation of −0.12‰. In the case of trichloromethane, an inverse isotope effect was even more pronounced by the fact that the lowest obtained ε value was 2.9‰, reaching even to a value of 4‰. This inverse isotope effect is consistent with the measured ε_C_ of 1.4 ± 0.2‰ by Horst and Lacrampe-Couloume^21^ and 1.5 ± 0.3‰ by Hunkeler et al.,^65^ but it does not agree with the finding reported here. Finally, isotope effects obtained for triethylamine within the range of 0.34–0.45‰ were overall in an excellent agreement with the experimentally observed ε value of 0.49‰.

#### 3.2 Explicit and Mixed Solvation Models

A more detailed solvent description that includes explicit interactions between the solvent and solute was applied by placing water molecules in proximity of the compounds under study. Clusters were prepared either manually or by selecting solvent molecules within a given range of intermolecular distances between them and the solute of interest out from a bigger water box. To also mimic bulk solvent effects, the constructed cluster models were additionally solvated in quantum-mechanical calculations by applying the PCM solvent model, or a full explicit solvation model was constructed.

##### 3.2.1 Benzene

In the case of the approach where explicit water-benzene models were prepared manually the resulting cluster models consisted of one, two, four, six, and eight water molecules. In the case of the smallest cluster model its two variants were prepared where the single water molecule was placed in the most likely locations and orientations to interact with benzene, either in an axial (over/below) or equatorial (on a side) position relative to the aromatic ring. Such structures were previously described in the literature.^29,66−69^ The cluster consisting of two water molecules was prepared with water molecules located in opposite axial positions, that is, one on each side of the aromatic ring. Then, more water molecules were added to the existing clusters, increasing their size. For bigger clusters—consisting of six and eight water molecules, each model was additionally preoptimized at the semiempirical theory level using the PM3 method to allow an additional solvent relaxation prior to the calculations at a target DFT level. Initial model structures are depicted in Figure S2. Calculated corresponding IE values, for calculations with and without additional implicit solvent model, are collected in Table 1.

**Table 1:** Carbon Isotope Effects (ε, ‰) for Equilibrium Water–Air Partitioning of Benzene Dissolved in Aqueous Solution Obtained from Calculations using Benzene-Water Clusters Prepared Manually with (Mixed) and without (Micro) Additional Continuum Solvent Model^a^

		DFT functional					
		B2PLYP-D3		B3LYP		M06-2X	
solvation model	No. of water molecules	smaller BS	larger BS	smaller BS	larger BS	smaller BS	larger BS
micro	**1**	–0.35	–0.22	0.18	0.03	0.25	0.06
	**1**_**side**_	–0.15		–0.15	0.02	–0.10	0.04
	**2**	–0.59	–0.32	0.26	0.02	0.27	–0.04
	**4**	–0.62	–0.27	0.23	0.09	0.33	0.07
	**6**	–0.87	–0.30	0.38	0.13	0.01	0.07
	**6**_**opt**_	–0.84	–0.32	0.15	–0.03	–0.03	–0.09
	**8**	–0.79		0.29		0.45	0.30
	**8**_**opt**_	–0.88		0.10	–0.05	–0.10	0.02
mixed	**1**	–0.22	0.04	0.46	0.30	0.40	0.38
	**1**_**side**_	0.36	0.03*	0.36	0.31	0.37	0.29
	**2**	–0.49		0.47	0.28	0.51	0.27
	**4**	–0.49	–0.08	0.44		0.65	0.14
	**6**	–0.54		0.41	0.27	0.57	0.12
	**6**_**opt**_	–0.55		0.42	0.26	0.28	0.01
	**8**	–0.46		0.45		0.16	
	**8**_**opt**_	–0.62		0.42	0.26	0.32	0.24

a Smaller BS, smaller basis set, 6-31+G(d,p); Larger BS, larger basis set, 6-311+G(2df,2p); opt–geometry preoptimized with the PM3 method; side–water molecule was located at a side of benzene aromatic ring; (*) water molecule initially located on the side of the benzene relocated above the aromatic system.

An additional set of microsolvation models was prepared by selecting water molecules located within different distances from the benzene molecule immersed in a water box. On the one hand, although initial model structures were prepared to contain up to 20 molecules, no successful calculations were completed for clusters of such size due to calculation convergence problems. Water molecules in clusters of smaller sizes, for which we were able to obtain results, on the other hand, were not distributed spherically around the solute, as one could expect, and therefore were not large enough to mimic an entire solvation shell around the solute (Figure S3). Nevertheless, isotope effect values obtained for benzene clusters with 5, 7, 9, and 12 water molecules are presented in Table 2.

**Table 2:** Carbon Isotope Effects (ε, ‰) for Equilibrium Water–Air Partitioning of Benzene Dissolved in Aqueous Solution Obtained from Calculations using Benzene-Water Clusters Prepared by Cutting out Solvent Molecules from Water Box, with (Mixed) and without (Micro) Additional Continuum Solvent Model^a^

		DFT functional					
		B2PLYP-D3		B3LYP		M06-2X	
solvation model	No. of water molecules	smaller BS	larger BS	smaller BS	larger BS	smaller BS	larger BS
micro	**5**	–0.77	–0.45	0.06	0.09	0.03	–0.19
	**7**	–0.95	–0.26	0.39	0.15	0.45	–0.26
	**9**			0.19	0.13	–0.05	0.26
	**12**				0.13	–0.04	
mixed	**5**	–0.43	–0.15	0.35	0.29	0.48	0.10
	**7**			0.42		0.40	0.14
	**9**					0.42	
	**12**					0.18	0.01

a Smaller BS, smaller basis set, 6-31+G(d,p); Larger BS, larger basis set, 6-311+G(2df,2p).

##### 3.2.1.1 Microsolvation Models

**Figure 3:**
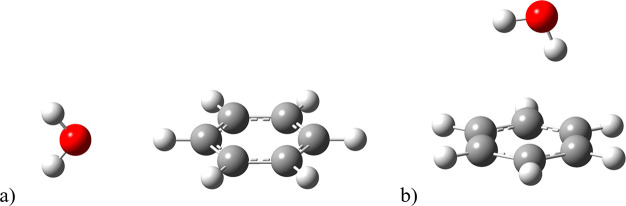
Geometries of benzene-water microsolvation models at the B2PLYPD3/6-31+G(d,p) theory level, where in the initial/final structures water was located (a) on a side (equatorial region) or (b) above/below the solute (axial region).

In the case of water-benzene cluster gas-phase calculations, that is, when only a few selected purely explicit solvent–solute interactions were taken into consideration in the solvent description, performed with the B2PLYP-D3 DFT functional, all results indicated a normal isotope fractionation regardless of the basis set used. The result closest to the experimental reference, ε of −0.12‰, was obtained for a cluster with only one water molecule located in an equatorial benzene region, in a way that the water oxygen atom was in front of one of the benzene hydrogen atoms (Figure 3a). In calculations performed with the smaller, 6-31+G(d,p), basis set the computed ε was −0.15‰. Other values obtained at this theory level ranged from −0.35‰, when the single water molecule was in the axial region of the aromatic benzene ring (Figure 3b), and from ca. −0.6 up to almost −0.9‰ for clusters consisting of more water molecules. In the case of calculations performed with the larger basis set, 6-311+G(2df,2p), the isotope fractionation was, in general, lower and less diverse than in the case of counterpart results obtained with the smaller basis set. Respective ε values were found within a range from −0.22‰, for the model with a single water molecule, to ca. −0.3‰ for the remaining systems, resulting in the average isotope fractionation value of ca. −0.3‰, the closest to the expected value among other theory levels tested.

An inspection of the vibrational modes obtained for the model with one water molecule that resulted in a very good match between theory and experiment revealed that modes that most contributed to the overall isotope effect do not change when a phase transfer occurred (Table S6). No contributing additional modes appear upon complex formation; hence, the observed and predicted effects are so small.

In contrast, when the two other DFT functionals were used the majority of calculated ε values did not agree even qualitatively with the measured isotopic fractionation; that is, they indicated an inverse isotopic fractionation. Only in a few cases calculations of water–benzene clusters reproduced an experimental, normal isotope effect of −0.12‰ quite well, especially in the case of the model consisting of a single water molecule that was located in a benzene equatorial region. Also, larger solvation models consisting of six and eight water molecules that were preoptimized prior to the target calculations using the M06-2X DFT functional resulted in an ε of ca. −0.1‰, depending on the basis set used.

The other approach in constructing initial benzene-water cluster models, in which solvent molecules were selected from a larger system, revealed a similar outcome as the results of calculations performed for the microsolvation models prepared manually. Calculations using the B2PLYP-D3 functional reproduced expected values qualitatively well, in all cases, and again the use of the larger basis set resulted in more moderate and, overall, more quantitatively correct isotope effect values. Using the M06-2X functional resulted in a mixture of normal and inverse isotopic fractionation—the latter ones especially for smaller models described with the larger basis set, while all results obtained with the B3LYP functional were qualitatively incorrect.

##### 3.2.1.2 Mixed Solvation Models

The incorporation of the continuum solvent model into benzene-water cluster calculations elevated isotope effects, and, as a result, only calculations for the models described with the B2PLYP-D3 functional resulted in the expected normal isotopic fractionation, except for the smallest systems consisting of a single water molecule placed manually near the benzene molecule. When calculations were performed with the larger basis set, both initial variants of this cluster, that is, with a water molecule placed either in the equatorial or axial region of the benzene ring, were optimized to the latter structure, and the results indicated a very small inverse isotope effect of only ∼0.03‰. Results from calculations using this DFT functional but combined with the smaller basis set were qualitatively correct in almost all cases but more pronounced. The only exception was the cluster model with a single water molecule placed manually in the benzene equatorial region, in a way the oxygen atom was oriented toward benzene hydrogen atoms, for which the obtained ε was 0.36‰. In contrast to calculations performed with the B2PLYP-D3 functional, the two remaining functionals applied to mixed solvation model calculations resulted only in qualitatively incorrect isotope effects, predicting an inverse isotopic fractionation upon benzene vaporization from water.

##### 3.2.1.3 Full Explicit Solvation Model

An all-atom representation of bulk solvation was used along with the QM/MM calculations within which the benzene molecule was treated quantum mechanically at the B2PLYP-D3/6-31+G(d,p) level of theory and 3096 water molecules were represented using the TIP3P potential (Table S7a). The obtained mean value of the isotope effect along with the computed standard error was 0.61 ± 0.08 and 0.33 ± 0.07‰ for 3N-6 and 3N frequencies, respectively, which does not agree with the measured very small but normal isotope effect.

A structural analysis of all 10 structures selected for isotope effect calculations revealed however that, in each of the selected structures, there is always at least one water molecule interacting directly with the aromatic ring at the distance of 1.97–2.55 Å like in the axial geometry of the benzene-water complex shown in Figure 3b.

The geometry of the system that resulted from the present DFT/MM calculations also agrees with a previously determined benzene-water hydrogen radial distribution function (BEN-H RDF),^29^ which depicted two peaks, the first one at ∼2.3 Å and the other one at ∼5 Å. It means that, up to the first minimum of BEN-H RDF (at 3.0 Å), one should expect a single hydrogen bond with benzene being a proton acceptor in the axial region of the molecule. Similar observations we could make based on the analysis of trajectories coming from classical molecular dynamics (MD) simulations performed on the benzene-water system prior to the DFT/MM optimizations.

Taking these observations into account we prepared another set of DFT/MM optimized structures in which the hydrogen-bonded water molecule was included in the QM region of the overall system (Table S7b). The predicted isotope effect improved significantly (ε = −0.18 ± 0.06 and −0.30 ± 0.06‰, resulting from the 3N-6 and 3N sets of vibrations, respectively). The positive effect of the presence of the nearby H-bonded water molecule is also seen in the distance between the water proton and the closest benzene carbon, which is 2.65 ± 0.26 Å.

##### 3.2.2 Trichloromethane

Trichloromethane-water solvation models were prepared manually not only with a goal to reproduce the most likely, if any, interaction of a water molecule with the solute hydrogen atom but also to study other possibilities by which the water environment could interact with electronegative chlorine atoms. Thus, two clusters, consisting of one and four water molecules, were prepared (Figure S4). The latter model was additionally preoptimized using the PM3 Hamiltonian. Predicted isotope effects are given in Table 3. In addition, trichloromethane-water clusters were prepared by selecting solvent from a water box surrounding the solute. The number of water molecules in solvation models prepared this way, and for which we were able to obtain converged results, ranged from 5 to 14. Their initial geometries are depicted in Figure S5. Corresponding isotope effect values are collected in Table 4. Finally, a large model consisting of ∼10 000 atoms was also used to capture both specific and bulk effects of the solvent (Table S7a,b). Considering that two recent studies on the subject provided an inverse carbon isotope effect and additionally agree with each other almost perfectly, we will use those values instead of the ones measured within this work as a reference for the results from the computational studies.

**Table 3:** Carbon Isotope Effects (ε, ‰) for Equilibrium Water–Air Partitioning of Chloroform Dissolved in Aqueous Solution Obtained from Calculations Using Chloroform–Water Clusters Prepared Manually with (Mixed) and without (Micro) Additional Continuum Solvent Model^a^

		DFT functional					
		B2PLYP-D3		B3LYP		M06-2X	
solvation model	No. of water molecules	smaller BS	larger BS	smaller BS	larger BS	smaller BS	larger BS
micro	**1**	0.33	0.31		0.55	–0.15	–0.14
	**4**	0.43	0.25	0.44	0.39	0.32	–0.33
	**4**_**opt**_	0.43	0.25	1.19	0.39	0.05	0.21
mixed	**1**	4.14	4.19	4.6	4.19	3.34	2.77
	**4**	3.98	3.59	3	3.54	3.2	2.4
	**4**_**opt**_	3.9		4.38	4.05	3.16	2.67

a Smaller BS, smaller basis set, 6-31+G(d,p); Larger BS, larger basis set, 6-311+G(2df,2p); opt—geometry preoptimized with the PM3 method.

**Table 4:** Carbon Isotope Effects (ε, ‰) for Equilibrium Water–Air Partitioning of Chloroform Dissolved in Aqueous Solution Obtained from Calculations Using Chloroform–Water Clusters Prepared by Cutting Out Solvent Molecules from Water Box with (Mixed) and without (Micro) Additional Continuum Solvent Model^a^

		DFT functional					
		B2PLYP-D3		B3LYP		M06-2X	
solvation model	No. of water molecules	smaller BS	larger BS	smaller BS	larger BS	smaller BS	larger BS
micro	**5**	0.51	0.58	1.73	1.61	–0.27	–0.26
	**7**	0.79	0.79	1.26	1.85	0.28	0.03
	**8**	0.74	0.9	1.23		0.67	
	**10**	0.95			0.82	0.54	
	**12**	0.89			1.18	0.3	
	**14**	0.65			1.33	1.23	
mixed	**5**	3.51	3.82	4.22	4	2.68	2.59
	**7**	3.73		4.17		2.84	2.66
	**8**	3.47		4.01		2.63	
	**10**	3.15			4.14		
	**12**					2.73	
	**14**	3.47				2.49	

a Smaller BS, smaller basis set, 6-31+G(d,p); Larger BS, larger basis set, 6-311+G(2df,2p).

##### 3.2.2.1 Mixed Solvation Models

The first thing one can notice is that the ε values obtained for the mixed solvation approach are within a range from 2.4 to ∼4.6‰. This is consistent with the results obtained for the pure PCM calculations and presents overestimated magnitudes of isotope effects.

##### 3.2.2.2 Microsolvation Models

When a continuum solvation model was not used in quantum-mechanical calculations, that is, solvation was represented only in an explicit way, isotopic fractionation was much less pronounced, and, in turn, the results were significantly closer to the experimental reference. Agreement with the experimental data was obtained in isotopic calculations for practically only one model prepared manually with four water molecules and preoptimized prior DFT calculations (4_opt_) treated with the B3LYP functional combined with the smaller basis set, which resulted in ε of 1.19‰, and almost the entire set of models prepared by cutting out the water molecules from a larger water system modeled also using B3LYP and both basis sets. For these models, the closest values of IEs to the experimental ones are within the range of 1.18–1.85‰. Remaining B3LYP models as well as two other DFT functionals used in this study resulted in the isotopic fractionation below 1‰, which also agrees with the experimental reference data but to a lesser extent. The exceptions are the smallest microsolvation models described with the M06-2X functional that provided a normal isotope effect.

Analysis of the C–H stretching vibration in a model mimicking an aqueous environment and in a gas-phase molecule revealed that the bond gets stronger upon transfer from water to air. This effect alone might be an indication of inverse isotope effects on elements involved in this stretching mode. However, if a complex is formed (or hydrogen bonds form), the number of vibrational modes increases, and the intermolecular vibrations, in particular, those originating from directed hydrogen bonds, will be additionally coupled to the C–H stretching mode. There is yet another vibrational mode that involves the motion of the hydrogen atom—the C–H bending mode, which along with the C–H stretching modes gives a combination mode arising from anharmonic coupling (see ref (70) and refs therein). These combination bands appear in the near-IR region, which is usually not covered in a normal mode analysis performed to compute isotope effects. Furthermore, in the common approach anharmonicity is neglected in those computations. Most of the studies related to this issue have been performed for pure liquid chloroform, which does not bring additional complications coming from the formation of hydrogen bonds in the structure. Those, however, should be considered when chloroform dissolved in aqueous solution is of interest. Rutkowski et al. have demonstrated the strengthening of the force constant of the C–H bending mode due to a H-bonded complex formation of chloroform and trimethylamine.^71^ Therefore, in principle, one might expect two opposite effects on the chloroform hydrogen atom motions upon complex formation: decreased frequency of the C–H stretching mode due to interactions with surrounding water molecules and increased frequency of the C–H bending mode. On the basis of the measured inverse C isotope effects on air–water partitioning of chloroform in this work and studies of others discussed herein^21,65^ one could conclude the former effect will be stronger and thus responsible for the observed direction of hydrogen isotopic fractionation. A detailed analysis of the sets of vibrational modes obtained for ^12^CHCl_3_ and ^13^CHCl_3_ complexed with water molecules and in the gas phase revealed that, upon complex formation, there are indeed two additional modes contributing quite significantly to the overall effect. In contrast, among all those strongly contributing modes to the carbon isotope effect the C–H bending is the weakest one (Table S9).

##### 3.2.2.3 Full Explicit Solvation Model

In contrast to the aforementioned data the large B3LYP/6-31+G(d,p)/TIP3P model resulted in a much diminished but still inverse carbon isotope effect of 0.08 ± 0.14‰. A quite large standard error as compared to the value itself as well as a large standard deviation of 0.46‰ clearly indicate a substantial fluctuation of the isotope effect magnitudes. Indeed, 10 selected structures resulted in the whole range of values from −0.70 to 0.82‰. The results obtained for the 3N instead of 3N-6 vibrations turned out to be very similar—ε_C_ = 0.04 ± 0.15‰, standard deviation of 0.47‰, and the range from −0.77 to 0.79‰. It is not possible to correlate the magnitude of the effect and the length of the hydrogen bond between the solute hydrogen and water oxygen. We can find the structures with a very tight interaction of 1.9 Å giving rise to an inverse effect, and those with only a slightly increased H_TCM_···O_WAT_ distance resulted in a normal effect. The other thing is that, regardless of the direction, the predicted as well as the measured effect is very small, and the applied approach is not sufficiently accurate. We also tested the effect of the inclusion of one water H-bonded water molecule in the QM region for the chloroform system. The effect resulted from an expansion of the QM region where the isotope effect was 0.34 ± 0.09 for 3N-6 and 0.31 ± 0.09‰ for 3N vibrations, which points to the right direction although it represents a bit underestimated magnitude.

##### 3.2.3 Triethylamine

Triethylamine can adopt several possible conformations. Nevertheless, conformational landscape studies of TEA^72^ pointed out seven local minima. Our calculations of isotope effects of an air–water partitioning for triethylamine, Table S11, showed that most stable conformers do not differ significantly in the computed isotopic fractionation.

To reproduce the expected most stabilizing nonbonded interaction between solvent and triethylamine, in the case of the smallest solvation model prepared manually we placed a water molecule in such a way that its hydrogen atom was pointed toward an amine nitrogen lone electron pair. For bigger models, more water molecules were additionally added near the aliphatic groups. As a result, cluster solvation models consisting of one, four, and five water molecules (Figure S6) were prepared. Similarly to the cluster models prepared manually for the two former solutes described, bigger clusters were also preoptimized with the semiempirical PM3 Hamiltonian. The resulting isotope effects are collected in Table 5. Results of calculations performed with B2PLYP-D3/6-311+G(2df,2p) are given in parentheses, since calculations for only the smallest model were successfully completed at this theory level. Moreover, these results for micro- and mixed solvation descriptions were almost the same as the corresponding values obtained with the smaller basis set. An additional set of solvation models was prepared from the water box surrounding triethylamine—consisting of up to 20 molecules, but successful calculations were performed only for clusters with no more than nine of them. Their initial geometries and respective isotope effects are presented in Figure S7 and Table 6, respectively. Finally, large models containing more than 10 000 atoms altogether were also prepared.

**Table 5:** Carbon Isotope Effects (ε, ‰) for Equilibrium Water–Air Partitioning of Triethylamine Dissolved in Aqueous Solution Obtained from Calculations Using Triethylamine–Water Clusters Prepared Manually with (Mixed) and without (Micro) Additional Continuum Solvent Model^a^

		DFT functional				
		B2PLYP-D3	B3LYP		M06-2X	
solvation model	No. of water molecules	smaller BS	smaller BS	larger BS	smaller BS	larger BS
micro	1	–0.25/(−0.27)*	–0.06	–0.21	–0.21	–0.17
	4	–0.33	–0.19	–0.24	–0.32	
	4_opt_	–0.41	–0.24	–0.27	–0.59	–0.53
	5	–0.25	–0.16	–0.37	–0.51	–0.49
	5_opt_	–0.4	–0.17		–0.38	
mixed	1	0.01/(0.01)*	0.19	0.21	0.04	0.02
	4	0.07	0.19	0.19	0.04	
	4_opt_	–0.15	–0.02	–0.02	–0.12	–0.17
	5	0.06	0.19		0.07	
	5_ppt_	–0.18	0.03	–0.03		–0.29

a Smaller BS, smaller basis set, 6-31+G(d,p); Larger BS, larger basis set, 6-311+G(2df,2p); opt–geometry preoptimized with the PM3 method; (*) result obtained using larger basis set.

**Table 6:** Carbon Isotope Effects (ε, ‰) for Equilibrium Water–Air Partitioning of Triethylamine Dissolved in Aqueous Solution Obtained from Calculations using Triethylamine–Water Clusters Prepared by Cutting Out Solvent Molecules from Water Box with (Mixed) and without (Micro) Additional Continuum Solvent Model^a^

		DFT Functional					
		B2PLYPD3		B3LYP		M06-2X	
solvationmodel	No. of water molecules	smaller BS	larger BS	smaller BS	larger BS	smaller BS	larger BS
micro	**1**	–0.39	–0.38	–0.22	–0.21	–0.36	
	**2**	–0.35	–0.43	–0.27	–0.27	–0.43	–0.43
	**3**	–0.23	0.13	0.05		–0.22	0.02
	**4**	–0.24	–0.07	–0.23	–0.28	–0.07	–0.08
	**7**	–0.40		–0.21	–0.35	–0.21	–0.44
	**9**	–0.47		–0.20	–0.31	–0.64	–0.70
mixed	**1**	0.45	0.4	0.42	0.36	0.45	0.39
	**2**	0.37	0.37			0.41	0.32
	**3**	–0.12	–0.20	0.37	0.38	0.36	
	**4**	0.02	0.04	0.36		0.01	0.33
	**7**	–0.30		0.04	0.37	–0.11	–0.16
	**9**	–0.06			0.36	–0.17	–0.10

a Smaller BS, smaller basis set, 6-31+G(d,p); Larger BS, larger basis set, 6-311+G(2df,2p).

##### 3.2.3.1 Microsolvation Models

Most of the calculations for cluster models indicated a normal isotopic fractionation. There were only two exceptions, namely, for the cluster prepared by cutting the solvent molecules out of the larger water system, consisting of three water molecules, described with the B2PLYP-D3 functional combined with the larger basis set, as well as when the B3LYP/6-31+G(d,p) theory level was used, for which obtained isotope effects were 0.13 and 0.05‰, respectively. These were the only values in a qualitative agreement with the experimental reference value of 0.49‰, although underestimated.

##### 3.2.3.2 Mixed Solvation Models

The inclusion of a continuum solvent representation in trimethylamine-water cluster calculations increased all ε values, which, in turn, resulted in a mixture of normal and inverse isotope effects almost for each combination of theory level and solvent model tested.

For the models prepared manually, in general, solvation models for which geometries were preoptimized with the semiempirical method prior to the target DFT calculation resulted in almost only normal vapor pressure isotope effects (VPIEs), from very small ε values, that is, close to 0, for calculations performed with B3LYP and for the single water molecule cluster described with the B2PLYP-D3 functional, through the range from −0.18‰ up to −0.3‰ for the results obtained with the remaining DFT functionals. In contrast, results of calculations for models that were not subjected to the additional optimization steps indicated inverse IEs with the highest absolute ε value of ∼0.2‰ for the clusters consisting of one, four, and five water molecules described with the B3LYP functional. These results were, in fact, in the closest agreement, although slightly underestimated, with respect to the experimental reference value of 0.49‰. Calculations for the respective structures described with the two remaining DFT functionals resulted in much more moderate but still qualitatively correct ε values of only up to 0.07‰.

When triethylamine-water clusters were prepared by cutting out the solvent environment from a larger solvent system and the B3LYP functional was used in quantum-mechanical mixed solvent model calculations, all resultant isotopic fractionations were again in a qualitative agreement and, in general, closer to the expected reference than the results obtained for the models prepared manually. It is interesting to note that the values of isotope effects calculated for the smaller solvation models described with both remaining DFT functionals, consisting of one or two and, additionally, with three and four water molecules in the case of the M06-2X functional, were in a better agreement than the corresponding results obtained for manually prepared systems. Nevertheless, an increasing number of water molecules present in the solvent models described with these two DFT functionals shifted the obtained isotopic fractionation toward a normal isotopic fractionation leading, in turn, to qualitatively wrong observations.

##### 3.2.3.3 Full Explicit Solvation Model

Because of unsatisfactory findings obtained for microsolvation models and being aware of the importance of the specific interaction between TEA and the closest water molecule in its aqueous solution a large model containing fully explicit representation of the solvent was constructed (details in the Supporting Information). Similarly to benzene and TCM it was treated using the DFT/MM level of theory. The B3LYP/6-31+G(d,p)/TIP3P potential provided a water–air partitioning carbon effect of −0.46 ± 0.11‰, which similarly to the benzene system is opposite to the trend observed experimentally. Including six librational frequencies in the calculations of isotope effects led to the even more normal carbon isotope effect of −0.59 ± 0.11‰. All structures resulted from classical MD simulations that were subsequently selected and subjected to the QM/MM calculations were characterized by the specific N_TEA_···H_WAT_ hydrogen bond of 1.93 ± 0.11 Å on average. It corresponds quite nicely with the first peak of the N_TEA_···H_WAT_ radial distribution function (RDF) at 1.85 Å computed based on the classical MD simulations (Figure S8). Adding one water molecule to the QM part of the overall system did not lead as in the case of benzene and TCM to agreement with the measured direction of an isotope effect but almost unchanged effects (−0.49 ± 0.11 and −0.60 ± 0.11‰ for 3N-6 and 3N vibrations, respectively). Both mean values are accompanied by large standard deviations of 0.84‰. The N_TEA_···H_WAT_ hydrogen bond got stronger, as its mean value was 1.85 ± 0.08 Å. Apparently capturing the most important interaction in this system does not guarantee success in reproducing the experimental direction of an isotope effect. It is also possible that the isotope effect predicted in the described way is somehow masked by the effect of other water molecules treated by the empirical force field, and the overall isotope effect may be shielded by, most likely, an unfavorable effect of the background charges assigned to the rest of the surrounding water molecules.

## 4 Discussion

### Intuitive Interpretation of Observable Isotope Effects Based on Expected Intermolecular Interactions

On the one hand, the normal isotope effect of volatilization in benzene (i.e., light isotopologues escaped preferentially) is in agreement with two studies reporting that the delocalized π-electrons of the aromatic ring function as hydrogen-bond acceptors^73,74^ leading to strong *inter*molecular interactions in the condensed phase that are broken when the substance is evaporated into the gas phase. A carbon isotope fractionation during a volatilization of pure benzene, on the other hand, was previously observed to lead to an inverse isotope effect,^25^ which, in turn, can be explained by the fact that benzene is not able to form hydrogen bonds with itself but interacts via van der Waals forces.^75^ As a result, the contribution of electrostatics is heavily diminished, and benzene interacts mostly via dispersion forces.^69^ These observations are, therefore, consistent with expectations that the properties of the *sorbent* in the condensed phase are a crucial factor for isotope effects of partitioning.

The inverse carbon isotope effect in the volatilization of TEA can on the one hand be explained by the fact that the ethyl side chains undergo only van der Waals interactions with the surrounding water molecules leading to additional *inter*molecular vibrations. Further, it may be rationalized that hyperconjugation (σ* (C-H)_CH_2__ → *n*_N_), which stabilizes the molecule in the gas phase, is lost due to hydrogen bonding between water and the lone pair at the N atom of triethylamine leading to pronounced changes in *intra*molecular vibrations.^71,76^ A hyperconjugation in the gas phase would lead to looser C–H bonds (i.e., C–H bond vibrations of lower energy) and stiffer C–N bonds (i.e., C–N bond vibrations of higher energy), whereas the loss of hyperconjugation in water would lead to stiffer C–H bonds (i.e., C–H bond vibrations of higher energy) and looser C–N bonds (i.e., C–N bond vibrations of lower energy).^71,76^ The changes in *intra*molecular C–H bond vibrations (i.e., change to lower vibrational energies on volatilization) therefore contribute to the direction of a normal isotope effect, whereas the changes in *intra*molecular C–N bond vibrations (change to higher vibrational energies) contribute to an inverse isotope effect. Vibrations of C–N bonds more strongly involve the movement of the carbon atom than of C–H bonds (as reflected in the reduced mass μ_C–N_ of the C–N bond vibration, which more strongly changes on substitution of ^12^C by ^13^C than μ_C–H_). Hence, the inverse carbon isotope effect of the C–N bond vibrations may be rationalized to dominate the contributions from *intra*molecular vibrations adding up to the inverse carbon isotope effect induced by the *inter*molecular van der Waals interactions.

The significant inverse hydrogen isotope effect during the volatilization of the CHCl_3_/CDCl_3_ mixture from an aqueous solution is in contradiction to our hypothesis that trichloromethane is a weak hydrogen-bond donor that interacts via hydrogen bonding in water so that a normal hydrogen isotope effect on volatilization would be expected.^77^ A possible explanation for this counterintuitive result is given by Rutkowski et al.,^71^ who showed that the hydrogen bonding of trichloromethane elongates the C–H bond lowering the frequency of its stretching vibration. Therefore, the experimental results must again be rationalized by changes in *intra* rather than *inter*molecular vibrations: the strong frequency shift in *intra*molecular vibrations may be hypothesized to lead to an inverse isotope effect, which would need to superimpose the predicted normal isotope effect expected from changes in *inter*molecular vibrations.

Therefore, while most measured isotope effects agree with intuitive predictions, predictions of the prevailing isotope effect based on *inter*molecular interactions expected from a molecular structure may also be wrong (e.g., in the case of trichloromethane), because *intra*molecular interactions can dominate the overall effect. Hence intuitive predictions reached their limits, so we explored computational predictions for better insight.

### Insight from an Interaction Analysis in Computational Predictions

In this work we performed an interaction analysis using SAPT for models, for which calculated isotope effects were in the best quantitative agreement with the experimental data.

In the case of benzene these were explicit solvent models prepared manually, described with the B2PLYP-D3/6-311+(2df,2p) theory level. The results are presented in Table S8.

The strongest interactions were found between water molecules located at both sides of the aromatic ring in its axial regions. Here, each such solvent molecule alone, according to the SAPT analysis, stabilized the complex by ∼3.3 kcal·mol^–1^. This magnitude is in a very good agreement with previously reported values obtained from the high-level theoretical calculations as well as with reference data obtained based on experimental studies.^69,78,79^ Energies of ca. −3 kcal·mol^–1^ for this particular interaction were also reported in the literature.^80,81^ Such stabilization results from stronger interactions between the benzene π-electron cloud and partially positive hydrogen atom from the water molecule. This agrees perfectly with the picture of the π-cloud donating electrons to a water hydrogen, thereby playing the role of a hydrogen-bond acceptor. The strength of such an effect is also influenced by interactions of a given water molecule with other solvent moieties, if present, as well as its position with respect to the solute itself. Stabilizing effects of the remaining benzene-water interactions, where water molecules did not interact with electrons of the aromatic system directly, were, in general, lower, up to ca. −1.2 kcal·mol^–1^ with one exception for water that formed interactions with two benzene hydrogen atoms by exposing its oxygen lone electron pairs, the interaction of which was of −1.9 kcal·mol^–1^.

Interactions between water molecules and solvated benzene slightly differ in their nature. The most stabilizing factor among most solvent–benzene interactions is dispersion. Electrostatics is comparable to or lower—by up to 20%—than the dispersion, except for the cluster consisting of six water molecules, which was preoptimized at the semiempirical PM3 theory level, where more disturbed ratios of energy terms were found. For two of the water molecules *D*/*E* ratios were even higher, showing electrostatics of less than ∼30% of the dispersion. Induction was the least important contribution, being only ∼15% of the electrostatics, but for the most strongly interacting water molecules with the solute this ratio was elevated even up to 45%. Nevertheless, it is important to note that no such strong differences in overall water-solvent interactions were found among other water molecules, that is, those not interacting directly with the π-electron cloud to such an extent. This is mostly due to the exchange energy term, which counteracts the presence of stronger attractive forces. In the case of models that resulted in the inverse isotope effects (e.g., the model with one water molecule −1_side_ obtained at the PCM/B2PLYP-D3/6-31+G(d,p) level of theory, see Table 1) the contribution of all terms was much lower, and the total interaction energy was less than −2 kcal·mol^–1^.

In the case of chloroform, intermolecular interactions were calculated with SAPT for explicit solvation models described with the B3LYP functional and both basis sets, as these models performed best with respect to the calculated isotope effects (Table S10).

**Figure 4:**
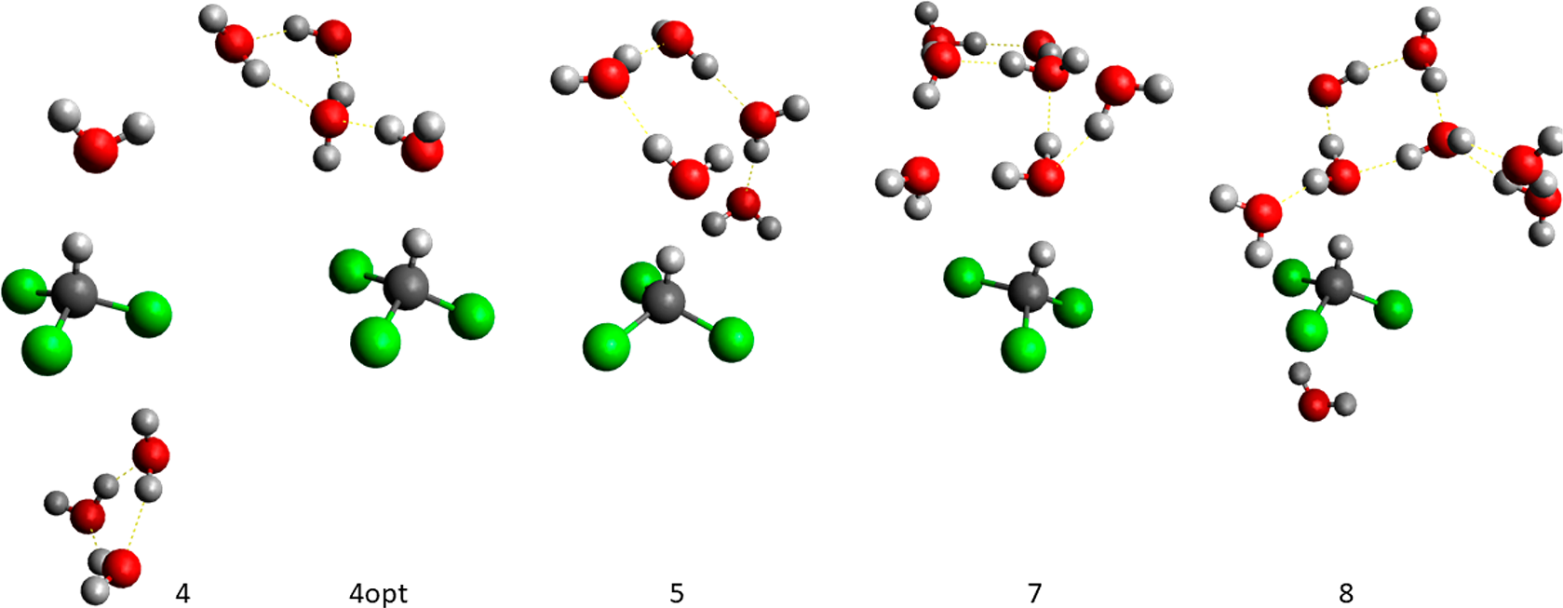
Geometries of trichloromethane-water microsolvation models resulted in the best agreement with the experimental values of IE and obtained at the B3LYP/6-31+(d,p) theory level. The numbers denote the number of water molecules in a model. “opt” denotes the preoptimization step with PM3 prior to DFT calculations.

The most significant stabilization of the solute was observed when a water molecule was positioned near the trichloromethane hydrogen atom—the stabilization effect obtained from the SAPT calculations was ∼4 kcal·mol^–1^, which was ∼3.5 times larger than for the effects of other water molecules found at the opposite side of the solute, that is, near the chlorine atoms (confer models 4 and 8, Figure 4). While the C–H bond alone is not likely to contribute significantly to any intermolecular nonbonding interactions, three highly electronegative chlorine atoms attached to the carbon atom in trichloromethane induce a significant polarization of the C–H bond, and as a result it is more prone to act like a proton donor when a lone electron pair of a water oxygen atom is found in its proximity. Consequently, the strongest interaction between the solvent and trichloromethane was dominated by the electrostatics, *D*/*E* ratio of ∼40%. In all models that resulted in the closest agreement with the experimental values of isotope effects (models 4opt, 5, 7, and 8, Figure 4) there was a strong, direct interaction established between the hydrogen atom of chloroform and the oxygen atom of a nearby water molecule. Water molecules not interacting with the solute hydrogen atom directly showed all sorts of *D*/*E* ratio values, including those that were negative, indicating the electrostatics were a destabilizing force in some cases, but their absolute energy contributions were small. Induction over the electrostatics ratio was within the range from ∼30 to 40% for all water molecules in all solvent models analyzed.

An interaction energy analysis performed on mixed solvent models of triethylamine-water treated with B3LYP/6-31+G(d,p) (Table S12) indicated that the most stabilizing interaction is when the solvent protons interact with the solute nitrogen atom. This interaction alone can stabilize the system by ∼10 kcal·mol^–1^, while other single water molecules provide a solvent–solute stabilization mostly only up to ca. −0.5 kcal·mol^–1^ for the models prepared from the large solvent system and up to ca. −1.4 kcal·mol^–1^ for the models prepared manually and preoptimized prior to the target DFT calculations. The latter effect was specific for water, which interacted by pointing one of its hydrogen atoms toward the oxygen of the water molecule directly involved in the interaction with the nitrogen atom, exposing its oxygen lone electron pair to the methyl group. The most important stabilizing factor for the strongest water-solute interaction with the nitrogen atom, if present, was the electrostatics. Induction and dispersion forces were ∼40% of that from electrostatics. Ratios between different types of interactions varied, in the case of other water molecules present in the solvent models, but their contributions were significantly smaller than was the case for this highly stabilizing water molecule, and in turn they contributed much less to the overall stabilization, especially for solvent being found at further distances from the aliphatic groups, for example; see structures depicted in Figure S9.

A deeper analysis of the smallest mixed model described with different DFT functionals confirmed the strong dependence of the closeness of the solvent water hydrogen atom to the triethylamine nitrogen for obtained isotope effects. A geometry optimization using the B3LYP functional located the nearest water hydrogen atom at the distance of 1.84 Å, while the two remaining functionals resulted in the respective distance below 1.81 Å.

A similar situation was encountered when respective clusters prepared manually obtained from two different optimization schemes were compared. When initial solvent model geometries were preoptimized with PM3 prior to the B3LYP calculations, a shortening of the distance between the nitrogen atom and water was observed. In these two cases, three hydrogen-bonded water molecules were always found at the nitrogen side due to more relaxation allowed, and the distance between the nitrogen atom and the water hydrogen atom was shortened to ∼1.81 Å. For these models, a shorter distance was accompanied by resulting isotope effects shifted toward a normal isotope effect.

## 5 Conclusions

In this work, we investigated the air–water partitioning of three different organic compounds dissolved in water by using experimental and theoretical approaches. Specifically, we demonstrated that a transfer of the studied organic compounds between two phases may be associated with a small but significant isotopic fractionation of a different direction and magnitude. The resultant isotope effects will depend on the nature and strength of noncovalent interactions between the organic solute and the water environment dominating in each of the studied systems. As a full, in-depth interpretation of the isotopic data was not possible based solely on the measured values of isotope effects, we extended the study by predicting isotope effects for the same systems using computational tools. Several different models were tested that varied in the way solvent effects were included as well as by the electronic structure method used to find a minimum energy structure and to calculate a force constants matrix to derive isotope effects. On the basis of the predicted values of isotope effects we selected the models that resulted in the best agreement with the measured values and considered them for the subsequent analysis of vibrational modes responsible for the observed isotope effects as well as of noncovalent interactions between each solute (benzene, chloroform, or triethylamine) and surrounding water molecules.

By looking closely at the vibrational modes for isotopically substituted and unsubstituted species we have found that, in the case of benzene, subtle changes in the respective magnitudes of the contributing modes upon phase transition can be responsible for the observed very small normal carbon isotope effect. The transfer of chloroform between the two phases, in contrast, resulted in a stronger interplay between various modes contributing to different directions resulting an overall small but inverse carbon isotope effect. The case of triethylamine is again different in this respect. As a branched molecule with carbon atoms that are bound directly and indirectly to the central nitrogen atom—where this nitrogen atom provides a free electron pair for the hydrogen bonding with a water molecule—different modes of the molecule are affected upon complex formation. What stands out is a highly localized concentration of modes around the substituted atom that contribute most to the overall isotope effect. This is a quite common observation for larger systems comprising several molecules. Apparently, the structure of triethylamine is prone to such behavior.

On the basis of the detailed analysis of the interaction energy, we have found that the most stabilizing forces for all studied systems are electrostatics and dispersion; however, their contribution differs in each of them. Dispersion plays the major role in stabilizing complexes of benzene and water. In contrast, for triethylamine-water solvation models, the dispersion energy term was significantly lower than the electrostatics. This is consistent with the poor performance of B3LYP functional for benzene-water models, in contrast to being a very good description for triethylamine-water systems. This DFT functional lacks the dispersion term, so in the theoretical description of solvent models the electron-rich solute molecule cannot interact “properly” with polar water molecules. We confirmed this finding by calculating additional isotope effects for benzene-water clusters described with the B3LYP functional together with the D3 Grimme’s dispersion correction, and the results, similarly to the results obtained with the B2PLYP-D3 functional, agreed with the experimental result (data not shown).

The lack of additional dispersion was favored in the theoretical description, however, when the two remaining solutes were studied, since the interaction was rather driven by the electrostatics. However, trichloromethane and triethylamine differ in a preferred type of solvent model. In the case of trichloromethane, the continuum solvent model does reproduce the experimental isotope effect qualitatively but overestimates its magnitude so that explicit interactions are necessary to properly describe the solvation of this polar compound. In the case of triethylamine, where three nonpolar groups can hinder the N atom exposition to the solvent, the continuum solvation model leads to satisfactory results. Possibly solvent–solute interactions described theoretically are overexpressed for triethylamine so that the best results for mixed solvent models were obtained with the B3LYP functional, which does not describe weak interatomic interactions well. This trend was reflected in the optimized clusters that were significantly less compact; that is, solvent molecules were found at further distances from the solute as well as from each other than in the corresponding models for which the other two DFT functionals were used for the geometry optimizations. Altogether, the interaction patterns found for chloroform in water tend to be in-between those for benzene and TEA. Its interactions with water molecules are stabilized rather by dispersion; however, electrostatics also plays a crucial role when it comes to contributions to the overall energy of the system.

It is worth mentioning that, when the interaction terms including dispersion were separated from those without (electrostatics, induction, exchange) and when the effect of either contribution on the overall stabilization of the systems was calculated, a significant role of dispersion was observed. A very similar finding was reported for other systems, like alcohols and hydrogen-bonded complexes that they can form.^82^

We conclude that it is crucial to choose an appropriate theory level carefully, allowing for a proper description of solvent–solute interactions—especially, when the investigated phenomenon is a result of weak intermolecular interactions, as in the case of the water–air equilibrium partitioning experiments modeled herein. Another factor that can affect theoretical findings is the system preparation. A supervised versus unsupervised approach in creating a solvent surrounding in proximity to a given solute can introduce different interactions, which may or may not be crucial for the outcome of the studies. This was the case, in particular, for solvating triethylamine, where an assumed likely solvent interaction with an amine group turned out to be incorrectly taken into an account in the theoretical description. Micro- or mixed solvent models also require increased attention when nonpolar and weakly polar compounds are solvated in water or any other polar solvent, since interactions between solvent and solute may be insufficiently taken into consideration due to a stronger interaction within the solvent itself. Finally, it is important to gain knowledge of which type of solvation model describes one’s system better. In our studies purely implicit models failed to reproduce the experimental trends either by providing the isotope effect of an opposite direction or heavily overestimated magnitudes. Only the triethylamine case was exceptional in this respect. The QM/MM models turned out to be quite reasonable for the benzene and the chloroform systems only when the QM part of the system comprised at least the water molecule responsible for forming a hydrogen bond with the solute molecule. In the case of triethylamine such an approach was not satisfactory at all. Its failure may have at least two sources: (i) insufficient size of the QM region prohibiting an accurate description of the direct nonbonding solute–solvent interactions, (ii) unfavorable interactions of the solutes with the water molecules described at the molecular mechanics level of theory. These issues require more complex models and more benchmarking studies, which are underway.

## Supporting Information Available

Lists The Supporting Information is available free of charge at https://pubs.acs.org/doi/10.1021/acs.jpcb.1c05574.

Details related to the analysis of the experimental data. Collected carbon isotope effects values calculated based on geometries, and Hessian calculations performed using PCM solvent model. Optimized geometries of models discussed in the study, figures depicting initial solvent models used in the study, and some explanatory examples of resulting geometries discussed in the text. Details of calculations using a full explicit solvation model. (PDF)
