# Supplementary material for: Isotope Effects on the Vaporization of Organic Compounds from an Aqueous Solution–Insight from Experiment and Computations
Source: J Phys Chem B. 2021 Dec 15;125(51):13868–85. doi: 10.1021/acs.jpcb.1c05574 (PMC8724799; doi:10.1021/acs.jpcb.1c05574)
Supplement: Supplementary file 1 — jp1c05574_si_001.pdf [file jp1c05574_si_001.pdf]

Supporting Information

for

Isotope Effects on the Vaporization of Organic Compounds from an Aqueous Solution – Insight from Experiment and Computations

Michał Rostkowski,[a] Heide K. V. Schürner,[b] Agata Sowińska,[a] Luis Vasquez,[a] Martyna Przydacz,[a] Martin Elsner,[b] and Agnieszka Dybala-Defratyka[a,*]

[a] *Institute of Applied Radiation Chemistry, Faculty of Chemistry, Lodz University of Technology, Zeromskiego 116, 90-924, Lodz, Poland*

[b] *Chair of Analytical Chemistry and Water Chemistry, Technical University of Munich, Elisabeth-Winterhalter-Weg 6, 81377 Munich, Germany*

[*] agnieszka.dybala-defratyka@p.lodz.pl

Theoretical Consideration to Exclude the Possibility of Deuterium Exchange in Partitioning Experiments with Chloroform

To assess the potential for deuterium exchange at room temperature and pH 7, we made use of literature data on alkaline hydrogen exchange of chloroform (control experiment in ref. 1). Assuming second-order kinetics with respect to chloroform and OH^-, a reported rate of 0.03 per hour at an aqueous OH^- concentration of 44 M would correspond to a rate of $7 \cdot 10^{-11}$ per hour (or $1.7 \cdot 10^{-9}$ per day) at an aqueous OH^- concentration of 10^{-7} M. However, this would be too slow by a factor of 10^7 to explain the observed changes in D/H of 10 per mille (or $1 \cdot 10^{-2}$) observed in the course of the experiment.

Full explicit solvation models

Calculations. Minimization was performed in five consecutive steps in which the force constant keeping the solute in place was varying from 500 to 50 $kcal \cdot mol^{-1} \cdot Å^{-2}$ using the steepest descent and the conjugate gradient methods. Next, the systems were heated from 100 to 300 K during 100 ps using the NVT ensemble with a time step of 1 fs and equilibrated using the NPT ensemble. The production runs were performed using the NPT ensemble with a time step of 1 fs and the length of the simulations was equal to 80 ns. Periodic boundary conditions with the particle mesh Ewald method[2] and 10 Å cut-off distance for nonbonding interactions were applied throughout the simulations. After classical MM MD simulations, 10 structures from each trajectory from the range of 20-80 ns were selected and subjected to QM/MM optimization at a DFT/MM level with the QM part consisting of all atoms of the solutes. Combination of a functional and basis set for the DFT/MM optimization was chosen based on the results obtained from the QM cluster calculations; for TCM and TEA it was B3LYP/6-31+G(d,p) and for benzene it was B2PLYP/6-31+G(d,p). The rest of the systems was described using the AMBER force field and the TIP3P potential implemented in fDynamo.[3] The optimization was performed as implemented in the fDynamo library[4] and its interface with Gaussian 09 (ver. C01 in case of TCM and E01 for benzene and TEA). Positions of all atoms outside 18 Å sphere were kept fixed during the geometry optimization. 10 Å cut-off distance for nonbonding interactions and 0.4 $kcal \cdot mol^{-1} \cdot Å^{-1}$ gradient tolerance were applied for the optimization purposes. Within every step

of the calculation a single-point gradient evaluation was performed, and the vibrational Hessian was determined for each properly converged structure.

Tables:

Table S1. Volume of stock solutions and different equilibration times of benzene, triethylamine, and trichloromethane for water-air and hexadecane-air partitioning, respectively.

	volume of stock solution [mL]			equilibration time [min]		
	**benzene**	**TEA**	**$CHCl_3$**	**benzene**	**TEA**	**$CHCl_3$**
**water-air**	2.80	0.07	1.00	30	60	30

Data Evaluation of Stepwise Partitioning

Data evaluation was carried out using both, the classical Rayleigh approach (eq. 8), and the following equations for stepwise partitioning as described in detail by Jeannottat *et al.*[5] In short, the following general equation applies for each equilibration step n

$$\delta\,^{h}E_{liquid,n+1} = \delta\,^{h}E_{liquid,n} \times f_{liquid} + \delta\,^{h}E_{HS,n} \times f_{HS} \qquad \text{(eq. S1)}$$

where f_{HS} and f_{liquid}, respectively, are the fractions of compound in the respective phase and $\delta^{h}E_{n+1}$ and $\delta^{h}E_{n}$ are the isotopic signatures before and after equilibration, respectively. Substitution of $\delta^{h}E_{liquid}$ by

$$\varepsilon \approx \delta\,^{h}E_{HS} - \delta\,^{h}E_{liquid} \qquad \text{(eq. S2)}$$

and rearrangement gives the isotopic shift after n equilibration steps (eq. S3)

$$\Delta\delta\,^{h}E_{HS} = n \times \varepsilon \times f_{HS} \qquad \text{(eq. S3)}$$

with

$$f_{HS} = \left(\frac{c_0 - c_{HS}}{c_0}\right)^{1/n} \qquad \text{(eq. S4)}$$

Enrichment factor ε can be determined as the slope of the linear regression of eq. S3.

Comparison of Rayleigh Data Evaluation with the Stepwise Partitioning Approach

Table S2. Comparison of data evaluation, *i.e.*, determination of enrichment factor ε, for benzene, triethylamine (TEA), and trichloromethane ($CHCl_3$) using Rayleigh equation (eq. 8) and the stepwise partitioning equation (eq. S1).

	**ε [‰]<br>Rayleigh equation**	**ε [‰]<br>stepwise partitioning**
**benzene, ε_C**	-0.12 ± 0.03 (R^2 = 0.62)	-0.11 ± 0.06 (R^2 = 0.25)
**TEA, ε_C**	0.49 ± 0.10 (R^2 = 0.72)	0.79 ± 0.18 (R^2 = 0.71)
**$CHCl_3$, ε_C**	-0.35 ± 0.14 (R^2 = 0.38)	-0.77 ± 0.61 (R^2 = 0.14)
**$CHCl_3$, ε_H**	1.79 ± 0.25 (R^2 = 0.80)	1.11 ± 0.56 (R^2 = 0.35)

Table S3. Isotope effects (ε, ‰) on benzene vaporization from aqueous solution obtained from calculations performed with PCM implicit solvent model.

**Method/Functional**	**Basis set**	**ε**
B3LYP	6-311++G(2d,2p)	**0.18**
B3LYP	6-31G(d,p)	**0.24**
M06-2X	6-31G(d,p)	**0.18**
MP2	6-311++G(2d,2p)	**0.20**
MP2	6-31G(d,p)	**0.27**
B3LYP	6-311+G(2df,2p)	**0.22**
M06-2X	6-31+G(d,p)	**0.43**
HF	6-311+G(2df,2p)	**0.24**
HF	6-31+G(d,p)	**0.47**
MP2	6-311+G(2df,2p)	**0.23**
MP2	6-31+G(d,p)	**0.44**
B3LYP	6-31+G(2df,2p)	**0.38**
B3LYP	6-311+G(2df,2p)	**0.19**
B3LYP	6-311+G(d,p)	**0.32**
B3LYP	6-311++G(d,p)	**0.17**
M06-2X	6-311+G(2df,2p)	**0.46**
M06-2X	6-311+G(d,p)	**0.47**
HF	6-31+G(2df,2p)	**0.39**
HF	6-311+G(2df,2p)	**0.16**
HF	6-311+G(d,p)	**0.47**
MP2	6-31+G(2df,2p)	**0.39**
MP2	6-311+G(2df,2p)	**-0.01**
MP2	6-311+G(d,p)	**0.27**
B3LYP	6-311+G(2df,2p)	**0.22**
B3LYP	6-31+G(d,p)	**0.37**
HF	6-311+G(2df,2p)	**0.24**
HF	6-31+G(d,p	**0.47**
MP2	6-311+G(2df,2p)	**0.23**
MP2	6-31+G(d,p)	**0.41**
B2PLYPD3	6-311+G(d,p)	**0.33**
B2PLYPD3	aug-cc-pvdz	**0.32**
B2PLYPD3	6-311+G(2df,2p)	**0.27**
B2PLYPD3	6-311++G(2df,2p)	**0.23**
B2PLYPD3	6-31+G(d,p)	**0.36**
M06-2X	6-311+G(d,p)	**0.47**
M06-2X	aug-cc-pvTZ	**0.15**
M06-2X	6-311+G(2df,2p)	**0.28**

M06-2X	6-31+G(d,p)	**0.46**
M11	aug-cc-pvtz	**0.01**
M11	6-311+G(2df,2p)	**0.32**
M11	6-311++G(2df,2p)	**0.20**

Table S4. Isotope effects (ε, **‰**) on trichloromethane vaporization from aqueous solution obtained from calculations performed with PCM implicit solvent model.

**Method/Functional**	**Basis set**	ε
B3LYP	6-311++G(2d,2p)	**3.85**
B3LYP	6-31G(d,p)	**3.79**
MP2	6-311++G(2d,2p)	**3.14**
B3LYP	6-31+G(2df,2p)	**3.76**
B3LYP	6-311+G(d,p)	**3.95**
B3LYP	6-311++G(d,p)	**4.02**
HF	6-31+G(2df,2p)	**3.58**
HF	6-311++G(d,p)	**3.81**
MP2	6-311+G(2df,2p)	**2.90**
MP2	6-311++G(d,p)	**3.07**
B3LYP	6-311+G(2df,2p)	**3.59**
B3LYP	6-31+G(d,p)	**3.83**
MP2	6-31+G(d,p)	**3.01**
B3LYP	6-311+G(2df,2p)	**3.60**
B3LYP	6-31+G(d,p)	**3.89**
MP2	6-311+G(2df,2p)	**2.96**
B2PLYP-D3	6-311+G(d,p)	**3.58**
B2PLYP-D3	aug-cc-pvdz	**3.46**
B2PLYP-D3	aug-cc-pvTz	**3.31**
B2PLYP-D3	6-311+G(2df,2p)	**3.34**
B2PLYP-D3	6-311++G(2df,2p)	**3.33**
M11	6-311++G(2df,2p)	**3.03**

Table S5. Isotope effects (ε, ‰) on triethylamine vaporization from aqueous solution obtained from calculations performed with PCM implicit solvent model.

**Method/Functional**	**Basis set**	**ε**
M06-2X	6-311++G(2d,2p)	**0.37**
M06-2X	6-31G(d,p)	**0.43**
HF	6-311++G(2d,2p)	**0.42**
B3LYP	6-31+G(2df,2p)	**0.38**
B3LYP	6-311++G(2df,2p)	**0.37**
B3LYP	6-311+G(d,p)	**0.38**
B3LYP	6-311++G(d,p)	**0.38**
M06-2X	6-31+G(2df,2p)	**0.39**
M06-2X	6-311++G(2df,2p)	**0.37**
M06-2X	6-311+G(d,p)	**0.38**
M06-2X	6-311++G(d,p)	**0.37**
HF	6-31+G(2df,2p)	**0.43**
HF	6-311++G(2df,2p)	**0.43**
HF	6-311+G(d,p)	**0.44**
HF	6-311++G(d,p)	**0.43**
MP2	6-31+G(2df,2p)	**0.39**
MP2	6-311++G(2df,2p)	**0.39**
MP2	6-311+G(d,p)	**0.40**
MP2	6-311++G(d,p)	**0.40**
B3LYP	6-311+G(2df,2p)	**0.37**
B3LYP	6-31+G(d,p)	**0.40**
M06-2X	6-311+G(2df,2p)	**0.37**
M06-2X	6-31+G(d,p)	**0.42**
HF	6-311+G(2df,2p)	**0.43**
HF	6-31+G(d,p)	**0.45**
MP2	6-311+G(2df,2p)	**0.39**
MP2	6-31+G(d,p)	**0.41**
B2PLYP-D3	6-311+G(d,p)	**0.41**
B2PLYP-D3	aug-cc-pvdz	**0.41**
B2PLYP-D3	6-311+G(2df,2p)	**0.40**
B2PLYP-D3	6-311++G(2df,2p)	**0.40**
B2PLYP-D3	6-31+G(d,p)	**0.43**
M06-2X	6-311+G(d,p)	**0.38**
M06-2X	aug-cc-pvdz	**0.38**
M06-2X	aug-cc-pvtz	**0.36**
M06-2X	6-311+G(2df,2p)	**0.37**
M06-2X	6-311++G(2df,2p)	**0.37**

M11	6-311+G(d,p)	**0.37**
M11	aug-cc-pvdz	**0.38**
M11	aug-cc-pvtz	**0.34**
M11	6-311+G(2df,2p)	**0.34**
M11	6-311++G(2df,2p)	**0.34**

Table S6. Normal modes (in cm^{-1}) obtained based on Hessian matrix calculation for $^{12}C_6H_6$ and $^{13}C_6H_6$ at the B2PLYP/6-311+G(2df,2p) level for the complex containing one water molecule (benzene-WAT) and the isolated molecule (benzene-air). Modes contributing the most to the overall isotope effect of -0.15 ‰ are shown in a bold font. The table presents the exemplary data for one (C1) out of six carbon atoms.

**benzene-WAT**			**benzene-air**		
**^{12}C ν**	**^{13}C ν**	**^{12}C ν /^{13}C ν ratio**	**^{12}C ν**	**^{13}C ν**	**^{12}C ν /^{13}C ν ratio**
**12C6H6**	**13C6H6**	**ratio**	**12C6H6**	**13C6H6**	**ratio**
18.8565	18.8098	1.0000	402.287	398.3914	0.9972
38.7018	38.6851	1.0000	402.3638	402.3603	1.0000
44.723	44.5051	1.0000	620.1915	613.6334	0.9932
97.8231	97.7222	1.0000	620.1955	618.9849	0.9987
127.7616	127.7462	1.0000	627.0078	623.2375	0.9960
248.2997	248.2977	1.0000	685.9679	685.6396	0.9996
400.4271	396.572	1.0028	857.7253	855.3757	0.9969
403.1982	403.1976	1.0000	857.7336	857.7333	1.0000
619.3238	612.7792	1.0068	959.9548	956.2065	0.9948
619.7902	618.5795	1.0013	959.9756	959.9756	1.0000
641.4977	637.7251	1.0040	960.1472	960.1235	1.0000
692.9	692.5478	1.0004	**1017.1815**	**1005.6085**	**0.9834**
864.6652	862.2553	1.0032	1020.462	1019.006	0.9979
866.4841	866.4836	1.0000	1068.33	1065.037	0.9951
969.2854	965.8833	1.0048	1068.352	1066.035	0.9966
969.5207	969.296	1.0003	1186.357	1185.774	0.9991
978.5597	978.2452	1.0004	1209.018	1206.896	0.9966
**1015.9176**	**1005.0124**	**1.0159**	1209.034	1209.022	1.0000
1020.968	1018.89	1.0030	**1381.3986**	**1371.0274**	**0.9827**
1067.388	1064.88	1.0037	1388.89	1388.022	0.9985
1068.228	1065.085	1.0047	**1525.614**	**1517.731**	**0.9863**
1187.514	1186.908	1.0010	1525.636	1522.631	0.9948
1209.337	1207.774	1.0025	**1653.0157**	**1638.9667**	**0.9751**
1209.93	1209.333	1.0009	1653.034	1649.928	0.9944
**1380.079**	**1369.8279**	**1.0173**	3208.454	3205.632	0.9941
1389.298	1388.318	1.0017	3218.165	3214.826	0.9931
**1524.47**	**1516.635**	**1.0138**	3218.193	3218.186	1.0000

1525.076	1522.09	1.0052	3233.576	3230.562	0.9937
1629.572	1629.555	1.0000	3233.605	3233.588	1.0000
**1650.0858**	**1637.2653**	**1.0232**	3243.209	3242.037	0.9976
1651.274	1646.988	1.0077	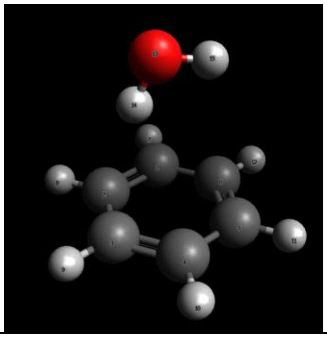		
3213.44	3211.137	1.0048			
3222.232	3220.487	1.0036			
3223.972	3222.278	1.0035			
3236.839	3235.377	1.0031			
3238.856	3236.878	1.0041			
3246.909	3245.67	1.0026			
3822.484	3822.484	1.0000			
3945.59	3945.59	1.0000			

Table S7a. Carbon isotope effects (ε, ‰) on water-air equilibrium partitioning obtained for the individual positions using the DFT/TIP3P models of benzene (BEN), chloroform (TCM), and triethylamine (TEA) aqueous solution (only the molecule of the respective solute was treated quantum mechanically) presented as a mean value with standard deviation.

	**BEN**		**TCM**		**TEA**		
	**Mean 3N-6/3N**	**Std.dev. 3N-6/3N**	**Mean 3N-6/3N**	**Std.dev. 3N-6/3N**	**Mean 3N-6/3N**	**Std.dev. 3N-6/3N**	
**C1**	0.45/0.20	0.62/0.54	0.08/0.04	0.46/0.47	-0.10/-0.18	0.74/0.74	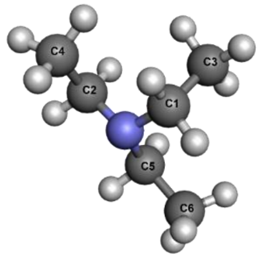
**C2**	0.37/0.13	0.79/0.73			-0.49/-0.59	0.24/0.23	
**C3**	0.55/0.32	0.67/0.62			-1.44/-1.57	1.34/1.35	
**C4**	0.75/0.44	0.59/0.55			-0.45/-0.63	0.69/0.66	
**C5**	0.93/0.58	0.44/0.54			-0.23/-0.32	0.66/0.67	
**C6**	0.62/0.32	0.35/0.37			-0.05/-0.25	0.44/0.42	

DFT for benzene - B2PLYP-D3/6-31+G(d,p), for TCM and TEA – B3LYP/6-31+G(d,p).

Table S7b. Carbon isotope effects (ε, ‰) on water-air equilibrium partitioning obtained for the individual positions using the DFT/TIP3P models of benzene (BEN), chloroform (TCM), and triethylamine (TEA) aqueous solution (the solute molecule and the water molecule forming a hydrogen bond with it were included in the QM part) presented as a mean value with standard deviation.

	**BEN**		**TCM**		**TEA**		
	**Mean 3N-6/3N**	**Std.dev. 3N-6/3N**	**Mean 3N-6/3N**	**Std.dev. 3N-6/3N**	**Mean 3N-6/3N**	**Std.dev. 3N-6/3N**	
**C1**	-0.23/-0.36	0.35/0.37	0.34/0.31	0.27/0.28	-0.08/-0.15	0.48/0.49	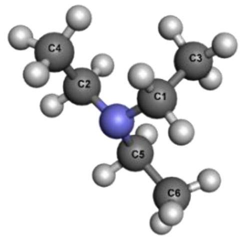
**C2**	-0.25/-0.37	0.41/0.45			-0.28/-0.37	0.25/0.25	
**C3**	-0.21/-0.32	0.53/0.58			-1.68/-1.79	1.17/1.17	
**C4**	-0.09/-0.20	0.49/0.51			-0.57/-0.70	0.50/0.52	
**C5**	-0.07/-0.19	0.55/0.58			-0.07/-0.16	0.63/0.64	
**C6**	-0.22/-0.35	0.50/0.53			-0.27/-0.44	0.46/0.44	

DFT for benzene - B2PLYP-D3/6-31+G(d,p), for TCM and TEA – B3LYP/6-31+G(d,p).

Table S8. Benzene-water interaction energy and its components computed by SAPT for microsolvation solvent models prepared manually, obtained at the B2PLYP-D3/6-311+G(2df,2p) theory level. Absolute interaction energies are given in kcal·mol^{-1}.

<table>
<tr><th rowspan="2">Total number of water molecules</th><th rowspan="2">No. of water mol.</th><th colspan="4">Absolute interaction energy contributions</th><th colspan="3">Ratios to electrostatics</th><th rowspan="3">Total SAPT energy</th></tr>
<tr></tr>
<tr><th></th><th></th><th>Electrostatics</th><th>Exchange</th><th>Induction</th><th>Dispersion</th><th>Exchange</th><th>Induction</th><th>Dispersion</th></tr>
<tr><td>1</td><td>1</td><td>-2.98</td><td>3.89</td><td>-1.06</td><td>-3.17</td><td>-1.31</td><td>0.36</td><td>1.07</td><td>-3.33</td></tr>
<tr><td></td><td></td><td></td><td></td><td></td><td></td><td></td><td></td><td></td><td></td></tr>
<tr><td>2</td><td>Total interaction</td><td>-5.75</td><td>7.31</td><td>-2.08</td><td>-6.12</td><td></td><td></td><td></td><td>-6.64</td></tr>
<tr><td rowspan="2">Per each water molecule present in a cluster computed separately</td><td>1</td><td>-2.87</td><td>3.65</td><td>-1.04</td><td>-3.06</td><td>-1.27</td><td>0.36</td><td>1.06</td><td>-3.32</td></tr>
<tr><td>2</td><td>-2.88</td><td>3.66</td><td>-1.04</td><td>-3.06</td><td>-1.27</td><td>0.36</td><td>1.06</td><td>-3.32</td></tr>
<tr><td></td><td></td><td></td><td></td><td></td><td></td><td></td><td></td><td></td><td></td></tr>
<tr><td>4</td><td>Total interaction</td><td>-9.66</td><td>13.26</td><td>-3.09</td><td>-9.93</td><td></td><td></td><td></td><td>-9.42</td></tr>
<tr><td rowspan="4">Per each water molecule present in a cluster computed separately</td><td>1</td><td>-2.89</td><td>3.71</td><td>-1.00</td><td>-3.14</td><td>-1.28</td><td>0.34</td><td>1.09</td><td>-3.32</td></tr>
<tr><td>2</td><td>-0.70</td><td>0.36</td><td>-0.07</td><td>-0.71</td><td>-0.51</td><td>0.10</td><td>1.01</td><td>-1.13</td></tr>
<tr><td>3</td><td>-2.65</td><td>3.68</td><td>-0.46</td><td>-2.42</td><td>-1.39</td><td>0.17</td><td>0.92</td><td>-1.86</td></tr>
<tr><td>4</td><td>-3.42</td><td>5.51</td><td>-1.55</td><td>-3.66</td><td>-1.61</td><td>0.45</td><td>1.07</td><td>-3.12</td></tr>
<tr><td></td><td></td><td></td><td></td><td></td><td></td><td></td><td></td><td></td><td></td></tr>
<tr><td>6</td><td>Total interaction</td><td>-7.95</td><td>12.24</td><td>-2.08</td><td>-10.32</td><td></td><td></td><td></td><td>-8.11</td></tr>
<tr><td rowspan="6">Per each water molecule present in a cluster computed separately</td><td>1</td><td>-0.84</td><td>0.50</td><td>-0.14</td><td>-0.89</td><td>-0.59</td><td>0.16</td><td>1.06</td><td>-1.37</td></tr>
<tr><td>2</td><td>-1.55</td><td>2.59</td><td>-0.23</td><td>-1.97</td><td>-1.67</td><td>0.15</td><td>1.27</td><td>-1.16</td></tr>
<tr><td>3</td><td>-0.79</td><td>0.61</td><td>-0.13</td><td>-0.79</td><td>-0.77</td><td>0.17</td><td>1.01</td><td>-1.10</td></tr>
<tr><td>4</td><td>0.15</td><td>0.34</td><td>-0.12</td><td>-0.73</td><td>2.25</td><td>-0.81</td><td>-4.82</td><td>-0.36</td></tr>
<tr><td>5</td><td>-3.39</td><td>5.66</td><td>-1.23</td><td>-3.99</td><td>-1.67</td><td>0.36</td><td>1.18</td><td>-2.94</td></tr>
<tr><td>6</td><td>-1.54</td><td>2.55</td><td>-0.23</td><td>-1.96</td><td>-1.65</td><td>0.15</td><td>1.27</td><td>-1.18</td></tr>
<tr><td></td><td></td><td></td><td></td><td></td><td></td><td></td><td></td><td></td><td></td></tr>
<tr><td>6_{opt}</td><td>Total interaction</td><td>-9.19</td><td>13.80</td><td>-2.98</td><td>-11.14</td><td></td><td></td><td></td><td>-9.50</td></tr>
<tr><td rowspan="6">Per each water molecule present in a cluster computed separately</td><td>1</td><td>-1.53</td><td>2.41</td><td>-0.23</td><td>-1.88</td><td>-1.58</td><td>0.15</td><td>1.23</td><td>-1.23</td></tr>
<tr><td>2</td><td>-1.25</td><td>2.02</td><td>-0.20</td><td>-1.74</td><td>-1.62</td><td>0.16</td><td>1.39</td><td>-1.16</td></tr>
<tr><td>3</td><td>-2.58</td><td>4.21</td><td>-1.12</td><td>-3.41</td><td>-1.63</td><td>0.44</td><td>1.32</td><td>-2.90</td></tr>
<tr><td>4</td><td>0.05</td><td>0.24</td><td>-0.09</td><td>-0.58</td><td>4.89</td><td>-1.87</td><td>-11.73</td><td>-0.38</td></tr>
<tr><td>5</td><td>-0.92</td><td>0.92</td><td>-0.24</td><td>-0.95</td><td>-1.01</td><td>0.26</td><td>1.04</td><td>-1.19</td></tr>
<tr><td>6</td><td>-2.96</td><td>3.99</td><td>-1.10</td><td>-2.58</td><td>-1.35</td><td>0.37</td><td>0.87</td><td>-2.65</td></tr>
</table>

opt – geometry preoptimized with the PM3 method.

Table S9. Normal modes (in cm^{-1}) obtained based on Hessian matrix calculation for $^{12}CHCl_3$ and $^{13}CHCl_3$ at the B3LYP/6-311+G(2df,2p) level for the complex containing three water molecules and the isolated chloroform molecule. Modes contributing the most to the overall isotope effect of 1.4 ‰ are shown in a bold font.

**$CHCl_3$-WAT**			**$CHCl_3$-air**		
**^{12}C ν**	**^{13}C ν**	**^{12}C ν /^{13}C ν ratio**	**^{12}C ν**	**^{13}C ν**	**^{12}C ν /^{13}C ν ratio**
9.900	9.899	1.0000	256.670	256.239	0.9998
36.832	36.803	1.0000	256.682	256.252	0.9998
43.431	43.427	1.0000	362.547	360.466	0.9986
51.563	51.526	1.0000	**663.049**	**645.252**	**0.9808**
59.617	59.559	1.0000	**730.574**	**707.826**	**0.9738**
69.390	69.281	1.0000	**730.674**	**707.921**	**0.9738**
137.434	137.434	1.0000	**1237.195**	**1230.304**	**0.9890**
140.800	140.774	1.0000	**1237.218**	**1230.326**	**0.9890**
152.480	152.478	1.0000	**3187.699**	**3166.408**	**0.9566**
162.893	162.890	1.0000			
188.162	188.162	1.0000			
196.767	196.766	1.0000			
223.784	223.783	1.0000			
254.552	254.101	1.0002			
256.391	255.994	1.0002			
288.689	288.685	1.0000			
330.663	330.463	1.0001			
366.788	365.045	1.0012			
517.660	517.608	1.0000			
**651.260**	**633.384**	**1.0193**			
**680.095**	**668.486**	**1.0130**			
682.064	677.948	1.0046			
**720.336**	**711.789**	**1.0100**			
**737.404**	**716.749**	**1.0245**			
**1304.407**	**1297.227**	**1.0118**			
**1329.702**	**1322.742**	**1.0116**			
1632.366	1632.366	1.0000			
1632.801	1632.800	1.0000			
1658.277	1658.275	1.0000			
**3077.117**	**3056.159**	**1.0444**			
3670.870	3670.853	1.0000			
3744.275	3744.275	1.0000			
3789.833	3789.833	1.0000			
3790.907	3790.907	1.0000			
3897.752	3897.752	1.0000			
3898.453	3898.453	1.0000			

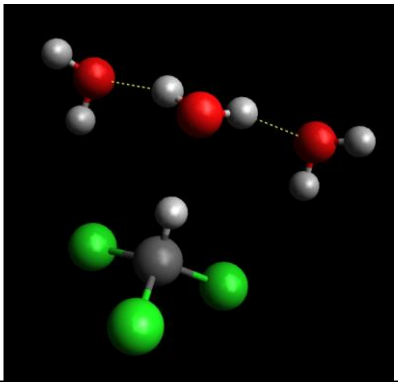

Table S10. Trichloromethane-water interaction energy and its components computed by SAPT for solvent models prepared manually and by cutting out solvent molecules from water box on microsolvation model structures obtained in calculations with the B3LYP DFT functional with smaller and larger basis set. 6-31+G(d,p) and 6-311+G(2df,2p), respectively. Absolute interaction energies are given in kcal·mol^{-1}.

**Total number of water molecules**	**No. of water mol.**	**Absolute interaction energy contributions**				**Ratios to electrostatics**			**Total SAPT energy**
		**Electrostatics**	**Exchange**	**Induction**	**Dispersion**	**Exchange**	**Induction**	**Dispersion**	
**Solvent models prepared manually**									
**6-31+G(d,p)**									
**4 opt**	**Total interaction**	-8.60	9.53	-2.61	-4.56				-6.24
Per each water molecule present in a cluster computed separately	**1**	-6.86	7.68	-1.95	-3.03	-1.12	0.28	0.44	-4.16
	**2**	-0.11	0.00	-0.01	-0.05	0.00	0.06	0.42	-0.16
	**3**	0.07	0.00	-0.01	-0.05	0.01	-0.09	-0.73	0.01
	**4**	-1.70	1.85	-0.64	-1.44	-1.09	0.38	0.85	-1.93
**Solvent models prepared by cutting out solvent molecules from water box**									
**5**	**Total interaction**	-11.90	14.68	-3.88	-6.22	-1.23	0.33	0.52	-7.32
Per each water molecule present in a cluster computed separately	1	-0.55	0.01	-0.03	-0.22	-0.02	0.05	0.40	-0.79
	2	-2.43	3.99	-1.23	-2.28	-1.64	0.51	0.94	-1.94
	3	-0.06	0.00	0.00	-0.05	-0.01	0.09	0.93	-0.11
	4	-8.31	10.68	-2.60	-3.55	-1.28	0.31	0.43	-3.78
	5	-0.55	0.00	-0.02	-0.12	-0.01	0.04	0.22	-0.69
**7**	**Total interaction**	-8.70	10.09	-2.64	-5.09	-1.16	0.30	0.58	-6.34
Per each water molecule present in a cluster computed separately	1	0.02	0.00	0.00	-0.03	0.00	-0.27	-1.72	-0.02
	2	-2.09	2.43	-0.79	-1.71	-1.16	0.38	0.82	-2.15
	3	-0.33	0.00	-0.02	-0.11	-0.01	0.06	0.32	-0.45
	4	-0.15	0.00	-0.01	-0.08	-0.01	0.07	0.56	-0.24
	5	-6.71	7.66	-1.80	-3.05	-1.14	0.27	0.45	-3.90
	6	0.15	0.00	0.00	-0.04	0.00	-0.03	-0.24	0.11
	7	0.42	0.00	-0.02	-0.08	0.00	-0.04	-0.19	0.32
**8**	**Total interaction**	-10.11	11.82	-3.11	-6.86	-1.17	0.31	0.68	-8.27
Per each water molecule present in a cluster computed separately	1	-7.35	8.69	-2.11	-3.20	-1.18	0.29	0.44	-3.97
	2	-0.55	0.01	-0.03	-0.15	-0.01	0.05	0.27	-0.71
	3	-1.27	1.74	-0.45	-1.81	-1.37	0.35	1.42	-1.79
	4	0.69	0.02	-0.04	-0.22	0.02	-0.06	-0.32	0.44

	5	-1.65	1.36	-0.46	-1.29	-0.82	0.28	0.78	-2.03
	6	0.14	0.01	-0.02	-0.13	0.06	-0.14	-0.93	0.00
	7	0.01	0.00	0.00	-0.04	0.00	-0.53	-4.24	-0.03
	8	-0.14	0.00	0.00	-0.03	0.00	0.03	0.24	-0.17
**6-311+G(2df,2p)**									
**5**	**Total interaction**	-11.57	14.07	-3.65	-6.33	-1.22	0.32	0.55	-7.47
Per each water molecule present in a cluster computed separately	1	-0.54	0.02	-0.03	-0.24	-0.03	0.06	0.44	-0.79
	2	-2.68	4.54	-1.29	-2.58	-1.69	0.48	0.96	-2.00
	3	-0.05	0.00	-0.01	-0.06	-0.01	0.10	1.16	-0.12
	4	-7.75	9.51	-2.30	-3.31	-1.23	0.30	0.43	-3.85
	5	-0.55	0.01	-0.02	-0.14	-0.01	0.04	0.26	-0.70
**7**	**Total interaction**	-12.91	16.00	-4.26	-7.55	-1.24	0.33	0.58	-8.73
Per each water molecule present in a cluster computed separately	1	-0.72	0.03	-0.04	-0.30	-0.05	0.06	0.42	-1.02
	2	-2.39	4.31	-1.28	-2.37	-1.80	0.53	0.99	-1.73
	3	-0.49	0.00	-0.03	-0.15	-0.01	0.06	0.30	-0.66
	4	-0.26	0.00	-0.02	-0.13	-0.02	0.06	0.50	-0.40
	5	0.10	0.01	-0.01	-0.13	0.11	-0.12	-1.35	-0.04
	6	-8.33	10.82	-2.57	-3.53	-1.30	0.31	0.42	-3.62
	7	-0.83	0.82	-0.32	-0.94	-0.99	0.39	1.14	-1.27
**10**	**Total interaction**	-6.46	6.66	-1.81	-4.60	-1.03	0.28	0.71	-6.21
Per each water molecule present in a cluster computed separately	1	-5.63	5.75	-1.50	-2.58	-1.02	0.27	0.46	-3.96
	2	0.84	0.24	-0.07	-0.59	0.29	-0.08	-0.71	0.42
	3	-0.51	0.60	-0.17	-0.85	-1.18	0.33	1.67	-0.92
	4	0.05	0.00	0.00	-0.02	0.00	-0.07	-0.44	0.02
	5	-0.65	0.06	-0.03	-0.31	-0.09	0.05	0.48	-0.93
	6	-0.13	0.00	0.00	-0.02	0.00	0.02	0.12	-0.15
	7	-0.09	0.00	-0.01	-0.11	-0.02	0.13	1.15	-0.21
	8	0.17	0.00	0.00	-0.03	0.00	-0.03	-0.17	0.14
	9	-0.49	0.00	-0.02	-0.08	0.00	0.04	0.16	-0.59
	10	-0.01	0.00	0.00	-0.01	0.00	0.20	1.61	-0.02
**12**	**Total interaction**	-7.37	7.91	-2.32	-5.18	-1.07	0.31	0.70	-6.96
Per each water molecule present in a cluster computed separately	1	-0.35	0.00	-0.01	-0.06	0.00	0.04	0.18	-0.43
	2	-6.05	6.49	-1.68	-2.71	-1.07	0.28	0.45	-3.95
	3	-1.01	1.05	-0.39	-1.05	-1.04	0.39	1.04	-1.40
	4	0.12	0.00	0.00	-0.02	0.00	-0.02	-0.16	0.10

	5	-0.13	0.00	0.00	-0.01	0.00	0.02	0.08	-0.15
	6	-0.20	0.00	-0.01	-0.04	0.00	0.03	0.18	-0.25
	7	0.09	0.00	0.00	-0.01	0.00	-0.03	-0.16	0.08
	8	0.89	0.08	-0.06	-0.39	0.09	-0.07	-0.43	0.53
	9	-0.05	0.00	-0.01	-0.04	0.00	0.11	0.90	-0.09
	10	-0.68	0.03	-0.04	-0.26	-0.04	0.06	0.37	-0.95
	11	0.09	0.00	-0.01	-0.06	0.01	-0.12	-0.69	0.02
	12	-0.09	0.27	-0.10	-0.53	-2.86	1.03	5.64	-0.45
**14**	**Total interaction**	-10.23	12.80	-3.64	-7.00	-1.25	0.36	0.68	-8.07
Per each water molecule present in a cluster computed separately	1	-6.73	7.67	-1.96	-2.94	-1.14	0.29	0.44	-3.96
	2	0.43	0.01	-0.04	-0.20	0.02	-0.09	-0.46	0.20
	3	-0.70	0.93	-0.30	-0.96	-1.33	0.42	1.37	-1.02
	4	0.02	0.00	0.00	-0.01	0.00	-0.03	-0.28	0.02
	5	-0.47	0.00	-0.02	-0.11	-0.01	0.04	0.24	-0.60
	6	0.02	0.00	0.00	-0.01	0.00	-0.05	-0.38	0.01
	7	-0.11	0.00	0.00	-0.03	0.00	0.02	0.25	-0.14
	8	-0.10	0.00	-0.01	-0.06	-0.01	0.08	0.63	-0.17
	9	0.04	0.00	0.00	-0.01	0.00	-0.03	-0.34	0.02
	10	-0.29	0.01	-0.01	-0.15	-0.02	0.05	0.52	-0.44
	11	0.06	0.00	0.00	-0.02	0.00	-0.03	-0.26	0.04
	12	-2.28	4.17	-1.28	-2.24	-1.83	0.56	0.98	-1.63
	13	-0.08	0.01	-0.01	-0.14	-0.11	0.16	1.67	-0.23
	14	-0.04	0.01	-0.01	-0.12	-0.19	0.29	3.13	-0.16

opt – geometry preoptimized with the PM3 method.

Table S11. Isotope effects for equilibrium partitioning of triethylamine dissolved in aqueous solution obtained from calculations performed at MP2/6-311++G(d,p) theory level using PCM implicit solvent model. Input structures we taken from potential energy surface analysis.[6]

Conformer		IE	
No.	Structure		ε
I	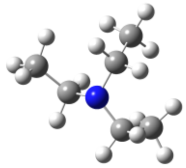	0.9966	0.44
II	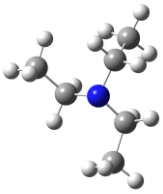	0.9966	0.40
III	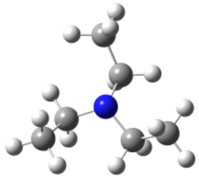	0.9966	0.42
IV	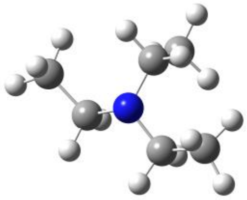	0.9966	0.44
V	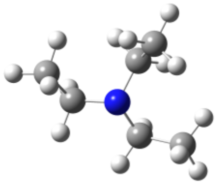	0.9966	0.42
VI	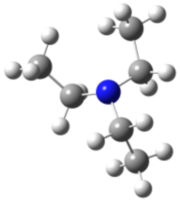	0.9967	0.29
VII		0.9964	0.56

Table S12. Triethylamine-water interaction energy and its components computed by SAPT for mixed solvent models described in calculations at the B3LYP/6-31+G(d,p) theory level. Absolute interaction energies are given in kcal·mol^{-1}.

**Total number of water molecules**	**Water No**	**Absolute interaction energy contributions**				**Ratios to electrostatics**			**Total SAPT energy**
**Smaller BS**		**Electrostatics**	**Exchange**	**Induction**	**Dispersion**	**Exchange**	**Induction**	**Dispersion**	
**1**	1	-17.28	21.87	-6.99	-7.06	-1.27	0.40	0.41	**-9.85**
**4**	**Total interaction**	-17.43	21.85	-7.02	-7.26				**-10.25**
Per each water molecule present in a cluster computed separately	1	-17.26	21.85	-6.98	-7.06	-1.27	0.40	0.41	**-9.85**
	2	-0.08	0.00	-0.02	-0.09	-0.02	0.20	1.10	**-0.19**
	3	0.00	0.00	0.00	-0.02	0.00	0.98	3.88	**-0.02**
	4	-0.08	0.00	-0.02	-0.09	-0.01	0.21	1.19	**-0.19**
**4_{opt}**	**Total interaction**	-18.64	23.65	-7.77	-8.58				**-11.76**
Per each water molecule present in a cluster computed separately	1	-18.37	23.36	-7.56	-7.12	-1.27	0.41	0.39	**-10.09**
	2	-0.85	0.15	-0.08	-0.60	-0.18	0.09	0.71	**-1.38**
	3	0.06	0.06	-0.06	-0.41	0.93	-0.99	-6.54	**-0.35**
	4	0.51	0.08	-0.06	-0.45	0.16	-0.13	-0.90	**0.06**
**5 wat chk**	**Total interaction**	-17.49	21.87	-7.03	-7.30				**-10.34**
Per each water molecule present in a cluster computed separately	1	-17.27	21.87	-6.99	-7.07	-1.27	0.40	0.41	**-9.85**
	2	-0.09	0.00	-0.02	-0.10	-0.02	0.23	1.16	**-0.21**
	3	0.00	0.00	0.00	0.00	0.00	0.06	0.27	**-0.01**
	4	-0.09	0.00	-0.02	-0.13	-0.03	0.26	1.34	**-0.24**
	5	-0.03	0.00	0.00	-0.01	0.00	0.03	0.30	**-0.04**
**5opt**	**Total interaction**	-	-	-	-				-
Per each water molecule present in a cluster computed separately	1	-	-	-	-	-	-	-	-
	2	-0.85	0.15	-0.09	-0.61	-0.17	0.11	0.72	**-1.41**
	3	0.01	0.00	0.00	-0.01	0.00	-0.08	-0.57	**0.00**
	4	0.53	0.03	-0.06	-0.36	0.06	-0.11	-0.68	**0.14**
	5	0.00	0.00	0.00	0.00	0.00	0.05	0.62	**-0.01**
**Structures prepared by cutting out solvent molecules from larger system**									
**1 water**	1	-0.14	0.17	-0.12	-0.60	-1.21	0.87	4.34	**-0.69**
**3**	**Total interaction**	-0.03	0.10	-0.13	-0.70				**-0.76**

Per each water molecule present in a cluster computed separately	1	0.02	0.00	0.00	-0.01	0.00	-0.17	-0.88	**0.00**
	2	0.00	0.04	-0.06	-0.29	15.51	-20.97	-100.55	**-0.30**
	3	-0.05	0.06	-0.07	-0.40	-1.10	1.35	7.61	**-0.46**
**4**	**Total interaction**	-0.22	0.25	-0.19	-1.18				**-1.34**
Per each water molecule present in a cluster computed separately	1	-0.08	0.14	-0.07	-0.41	-1.68	0.81	5.13	**-0.43**
	2	-0.11	0.06	-0.05	-0.30	-0.54	0.51	2.78	**-0.40**
	3	-0.02	0.00	-0.01	-0.08	-0.07	0.46	5.47	**-0.11**
	4	-0.01	0.05	-0.07	-0.38	-4.26	5.27	29.99	**-0.40**
**7**	**Total interaction**	-16.42	20.36	-6.71	-8.12				**-11.21**
Per each water molecule present in a cluster computed separately	1	-0.09	0.14	-0.06	-0.44	-1.44	0.68	4.63	**-0.46**
	2	-0.09	0.06	-0.05	-0.29	-0.63	0.56	3.31	**-0.37**
	3	-0.02	0.00	0.00	-0.01	0.00	0.07	0.48	**-0.03**
	4	-0.19	0.00	-0.01	-0.09	-0.01	0.05	0.47	**-0.29**
	5	0.10	0.04	-0.06	-0.35	0.42	-0.60	-3.38	**-0.26**
	6	0.54	0.05	-0.04	-0.37	0.08	-0.07	-0.68	**0.17**
	7	-16.67	20.08	-6.48	-6.57	-1.20	0.39	0.39	**-9.96**

opt – geometry preoptimized with the PM3 method.

Figures

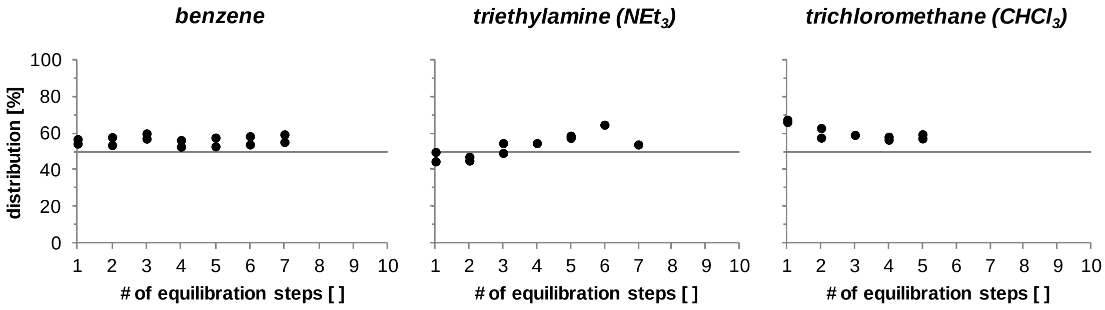

Figure S1. Distribution of benzene, triethylamine and trichloromethane during water-air partitioning. Points that lie above the 50:50 distribution line indicate higher proportion of substance into the gas phase. while points that lie below that line show higher proportion in the liquid phase.

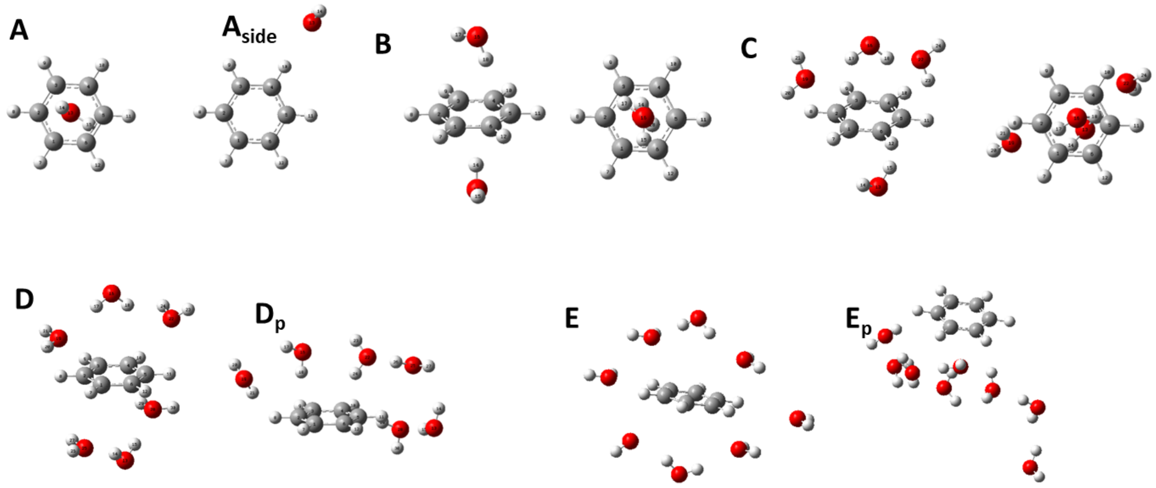

Figure S2. Initial geometries of benzene-water clusters prepared manually by placing water molecules in proximity to the solute. P – denotes structure after initial preoptimization with the PM3 method. Side – denotes structure in which water was in equatorial position. *i.e.* on a side of the benzene ring. Models A to E consist of 1, 2, 4, 6, and 8 water molecules. respectively.

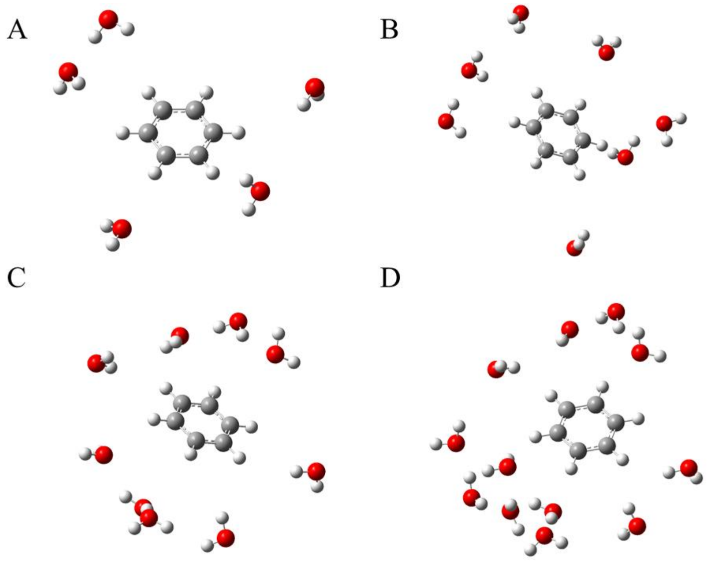

Figure S3. Initial geometries of benzene-water clusters prepared by cutting out solvent molecules from larger water box structure. Models A to D consist of 5, 7, 9, 12 water molecules. respectively.

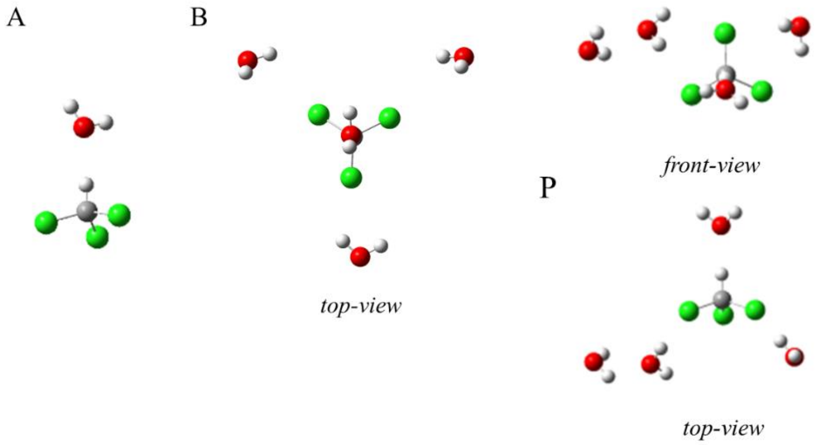

Figure S4. Initial geometries of trichloromethane-water clusters prepared manually by placing water molecules in proximity to the solute. Models A and B contain 1 and 4 water molecules, respectively. P – denotes structure after initial preoptimization with the PM3 method. For the latter front and top view is presented in respect to C-H trichloromethane bond.

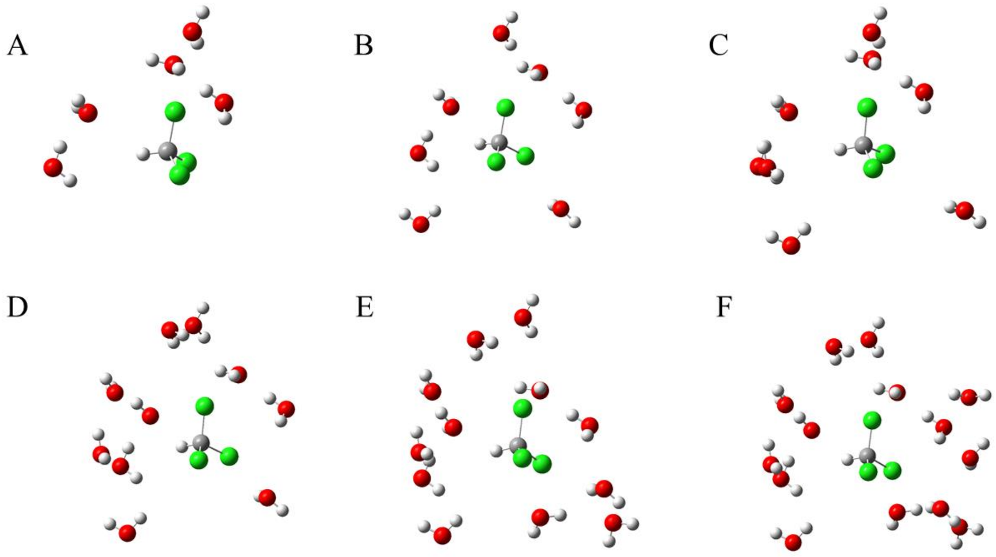

Figure S5. Initial geometries of trichloromethane-water clusters prepared by cutting out solvent molecules larger water box structure. Models A to F contain 5, 7, 8, 1, 12, and 14 water molecules, respectively.

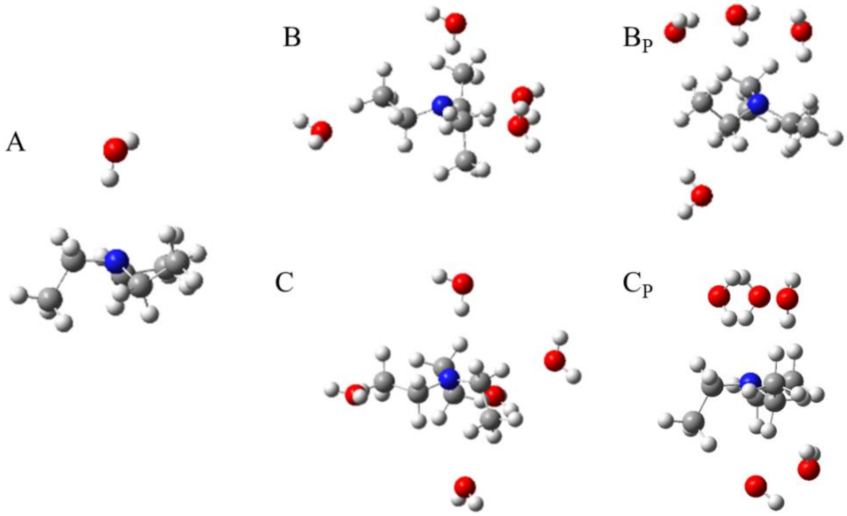

Figure S6. Initial geometries of triethylamine-water clusters prepared manually by placing water molecules in proximity to the solute. Models A. B and C contain 1, 4, and 5 water molecules, respectively. P – denotes structure after initial preoptimization with the PM3 method.

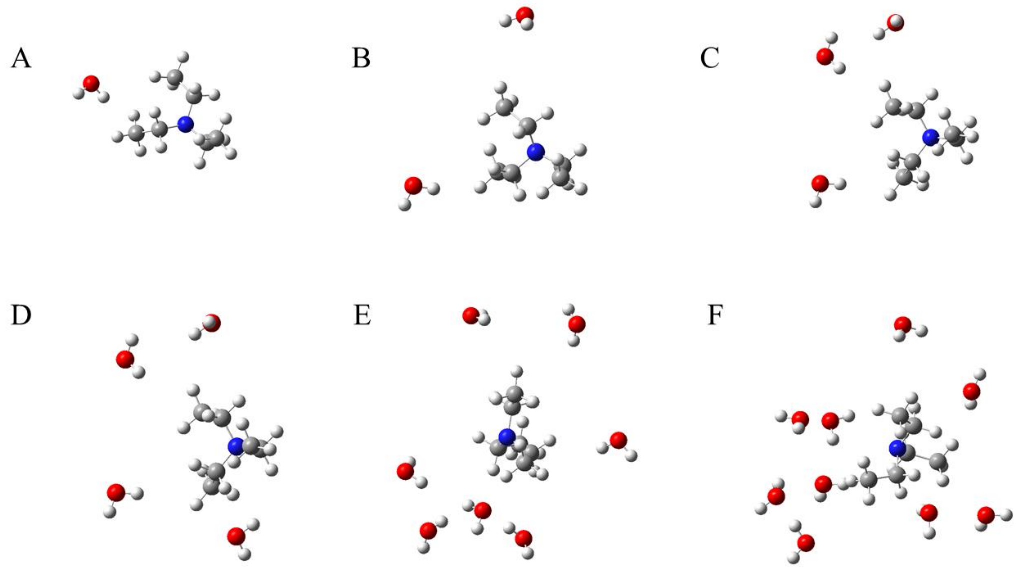

Figure S7. Initial geometries of triethylamine-water clusters prepared by cutting out solvent molecules from larger water box structure. Models A to F contain 1, 2, 3, 4, 7, and 9 water molecules, respectively.

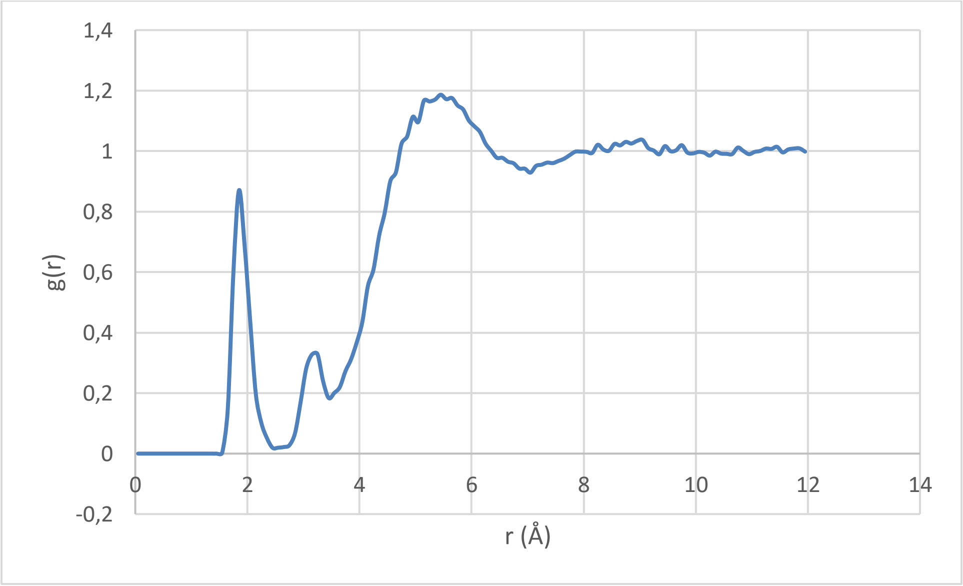

Figure S8. Radial distribution functions between the nitrogen of TEA and the one of the hydrogen atoms of water.

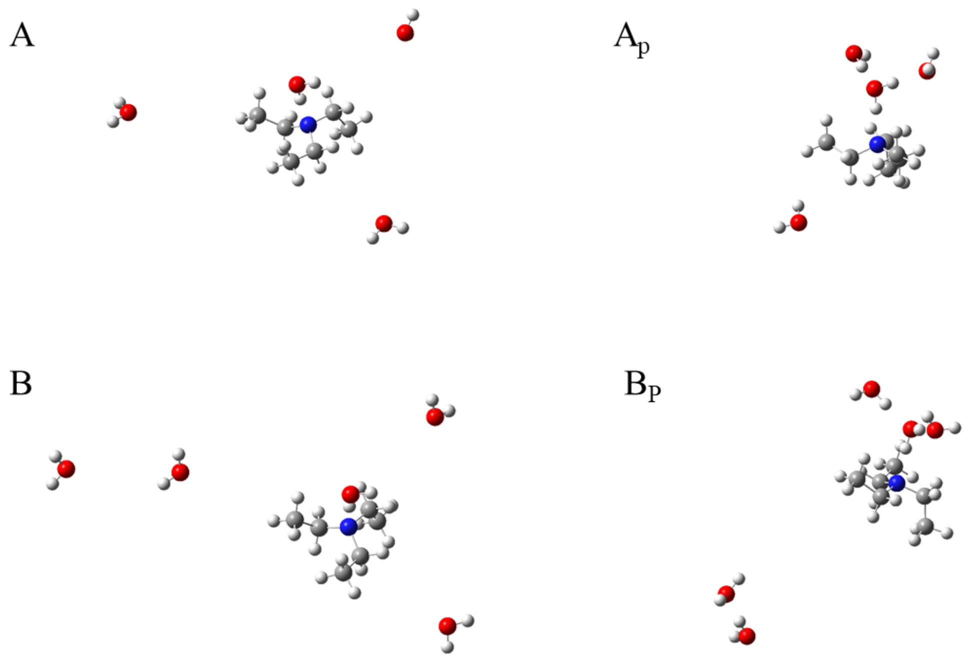

Figure S9. Geometries of triethylamine-water mixed solvent models on structures prepared manually. A and B consisting of 4 and 5 water molecules, respectively optimized in presence of PCM at B3LYP/6-31+G(d,p) theory level. P – structures obtained with preoptimization step with the PM3 method.

References:

(1) Higuera-Padilla, A. R.; Kock, F. V. C.; Batista, A. A.; Colnago, L. A. A Straightforward Catalytic Approach to Obtain Deuterated Chloroform at Room Temperature. *Magn. Reson. Chem.* **2020**, *58* (10), 917–920. https://doi.org/10.1002/mrc.5066.

(2) Darden, T.; York, D.; Pedersen, L. Particle Mesh Ewald: An N·log(N) Method for Ewald Sums in Large Systems. *J. Chem. Phys.* **1993**. https://doi.org/10.1063/1.464397.

(3) Krzemińska, A.; Paneth, P.; Moliner, V.; Świderek, K. Binding Isotope Effects as a Tool for Distinguishing Hydrophobic and Hydrophilic Binding Sites of HIV-1 RT. *J. Phys. Chem. B* **2015**, *119* (3), 917–927. https://doi.org/10.1021/jp506119h.

(4) Field, M. J.; Albe, M.; Bret, C.; Proust-De Martin, F.; Thomas, A. The Dynamo Library for Molecular Simulations Using Hybrid Quantum Mechanical and Molecular Mechanical Potentials. *J. Comput. Chem.* **2000**, *21* (12), 1088–1100. https://doi.org/10.1002/1096-987X(200009)21:12<1088::AID-JCC5>3.0.CO;2-8.

(5) Jeannottat, S.; Hunkeler, D. Chlorine and Carbon Isotopes Fractionation during Volatilization and Diffusive Transport of Trichloroethene in the Unsaturated Zone. *Environ. Sci. Technol.* **2012**, *46* (6), 3169–3176. https://doi.org/10.1021/es203547p.

(6) Nguyen, H. V. L.; Kannengießer, R.; Stahl, W. Microwave Survey of the Conformational Landscape Exhibited by the Propeller Molecule Triethyl Amine. *Phys. Chem. Chem. Phys.* **2012**, *14* (33), 11753–11758. https://doi.org/10.1039/C2CP41385J.

Geometries of optimized models used in the study

Benzene-Water cluster models prepared manually optimized at B2PLYP-D3/6-311+G(2df,2p) theory level.

Benzene - 1 Water

C	-0.60598	0.41240	1.33746
C	-0.36420	-0.93668	1.09108
C	-0.38288	-1.42031	-0.21505
C	-0.64354	-0.55478	-1.27417
C	-0.88673	0.79300	-1.02704
C	-0.86774	1.27656	0.27854
H	-0.58588	0.78814	2.35004
H	-0.15930	-1.60766	1.91271
H	-0.19185	-2.46627	-0.40652
H	-0.65285	-0.92929	-2.28738
H	-1.09016	1.46429	-1.84895
H	-1.05579	2.32321	0.46993
O	2.73272	0.25755	-0.11471
H	1.88712	-0.16212	0.07027
H	2.49332	1.10827	-0.48734

Benzene - 2 Water molecules

C	0.13870	1.16807	-0.71393
C	-0.00932	-0.05539	-1.36311
C	-0.14831	-1.22502	-0.61962
C	-0.14006	-1.17088	0.77278
C	0.00898	0.05198	1.42153
C	0.14874	1.22130	0.67843
H	0.24710	2.07516	-1.29046
H	-0.01638	-0.09701	-2.44221
H	-0.26417	-2.17382	-1.12303
H	-0.25427	-2.07753	1.34892
H	0.01656	0.09362	2.50077
H	0.26986	2.16965	1.18147
O	-3.39940	0.03096	-0.08529
H	-2.48698	-0.26267	-0.16253
H	-3.33237	0.92955	0.24323
O	3.40004	-0.02313	-0.08699
H	2.48650	0.26889	-0.15677
H	3.33662	-0.92876	0.22234

Benzene - 4 Water molecules

C	-0.94539	0.25616	-1.42592
C	-0.21673	-0.63129	-0.63965
C	-0.25390	-0.52343	0.74985
C	-1.02087	0.47335	1.35032
C	-1.74819	1.36539	0.56205
C	-1.70974	1.25639	-0.82650
H	-0.92330	0.16870	-2.50261
H	0.38794	-1.40518	-1.08777
H	0.32776	-1.21323	1.34252
H	-1.05653	0.55353	2.42723
H	-2.34616	2.13518	1.02809
H	-2.27869	1.94235	-1.43761
O	-3.77108	-1.53181	-0.02669
H	-3.15942	-2.25889	0.10592
H	-3.20338	-0.76004	-0.10813
O	3.64762	0.09890	-0.37155
H	3.85817	0.20592	-1.30069
H	3.06457	0.84469	-0.14696
O	2.35688	-2.32709	0.26385
H	3.00797	-2.95787	0.57444
H	2.85836	-1.51792	0.06344
O	1.80491	2.09140	0.38548
H	0.99518	1.59967	0.57068
H	1.52984	2.83250	-0.15820

Benzene - 6 Water molecules

C	1.16263	-1.11094	-1.13292
C	0.49399	-1.08903	0.08771
C	1.19633	-0.90806	1.27521
C	2.57885	-0.74083	1.24073
C	3.25312	-0.75651	0.02051
C	2.54493	-0.94420	-1.16563
H	0.59640	-1.24356	-2.04315
H	-0.57870	-1.19603	0.11092
H	0.65689	-0.88213	2.21064
H	3.12989	-0.59598	2.15911
H	4.32567	-0.62556	-0.00562
H	3.06969	-0.95611	-2.11033
O	-0.91025	2.28193	1.18109
H	0.03644	2.27783	0.92028
H	-1.03248	3.03886	1.75772
O	-2.03654	-0.70142	-2.29491
H	-2.50697	-1.01469	-1.51550
H	-1.72520	0.18272	-2.05958
O	-3.23803	-1.81451	0.23090
H	-4.18932	-1.94415	0.26356
H	-2.85460	-2.68743	0.35335
O	-1.08959	1.98909	-1.58986
H	-1.31321	2.20018	-0.66685

H	-1.53465	2.64361	-2.13227
O	1.44809	2.23325	-0.16476
H	0.92383	2.07941	-0.96256
H	1.98038	1.43659	-0.05667
O	-1.96105	-0.16790	2.40311
H	-1.60851	0.63228	1.99172
H	-2.45588	-0.61197	1.70694

Benzene - 6 Water molecules preoptimized with PM3

C	-3.13808	-0.31642	0.62179
C	-3.08773	-0.18376	-0.76436
C	-1.85715	-0.17371	-1.41868
C	-0.67719	-0.29094	-0.68861
C	-0.72757	-0.42093	0.69836
C	-1.95716	-0.43716	1.35236
H	-4.09221	-0.32688	1.12904
H	-4.00239	-0.08975	-1.33174
H	-1.81833	-0.06690	-2.49296
H	0.27979	-0.26606	-1.18900
H	0.19284	-0.50269	1.25585
H	-1.99502	-0.53497	2.42794
O	2.39638	-1.78640	0.32352
H	2.51545	-1.37345	-0.54046
H	1.72342	-2.47032	0.20177
O	1.26958	2.45099	0.15580
H	0.31807	2.62923	0.12312
H	1.49587	2.04411	-0.68789
O	-1.57350	2.92834	0.08382
H	-2.01669	2.07441	0.03648
H	-1.93124	3.35809	0.86375
O	3.29717	0.78759	1.17318
H	2.52728	1.37807	1.18191
H	2.94705	-0.10671	1.29058
O	2.81934	0.48278	-1.48753
H	3.21863	0.66370	-0.60791
H	3.50461	0.65244	-2.13670
O	0.10848	-3.49657	-0.09737
H	-0.57103	-2.82275	0.01065
H	-0.16638	-4.23593	0.44902

Trichloromethane-Water cluster models prepared manually optimized at B3LYP/6-31+G(d,p) theory level.

Trichloromethane - 4 Water molecules preoptimized with PM3

C	1.85903	0.00995	0.05501
H	1.18349	0.05654	0.91039
Cl	2.78065	-1.51733	0.14944
Cl	0.83085	0.03723	-1.43330
Cl	2.93951	1.42862	0.09530
O	-0.39653	0.09271	2.17733
H	-1.21911	0.01629	1.65263
H	-0.55684	-0.33839	3.02426
O	-4.63587	1.42302	-0.04065
H	-4.98474	2.06712	-0.66735
H	-5.08398	0.56940	-0.21064
O	-4.85952	-1.30459	-0.37295
H	-5.24334	-2.02142	0.14528
H	-3.90894	-1.26269	-0.15260
O	-2.36178	-0.07909	0.18738
H	-1.70299	-0.01191	-0.51950
H	-2.97531	0.68412	0.08401

Trichloromethane-Water cluster models prepared by cutting out solvent molecules from water box optimized at B3LYP/6-31+G(d,p) theory level.

Trichloromethane - 5 Water molecules

C	-1.72300	-0.26990	-0.06320
H	-0.75927	-0.58135	-0.48192
Cl	-2.79610	-1.68232	0.07458
Cl	-1.38054	0.42427	1.57771
Cl	-2.42308	0.98584	-1.13067
O	2.83027	1.45353	-0.84935
H	2.19229	2.02975	-0.36839
H	3.18371	1.96425	-1.58784
O	0.91535	2.78808	0.60297
H	0.36638	3.50238	0.25527
H	0.29537	2.12186	0.94348

O	4.53294	-0.32260	0.48825
H	3.99182	0.38642	0.08196
H	4.88720	0.03013	1.31237
O	1.03583	-0.87939	-1.12758
H	1.60799	-1.59914	-0.78973
H	1.63221	-0.11891	-1.22660
O	3.01085	-2.58771	-0.05758
H	2.88974	-3.20213	0.67508
H	3.64379	-1.90158	0.24430

Trichloromethane - 7 Water molecules

C	-2.49961	-0.16832	0.27401
H	-1.62719	0.48777	0.29526
Cl	-3.04756	-0.45959	1.94423
Cl	-3.76746	0.61927	-0.70737
Cl	-1.97029	-1.71862	-0.49731
O	4.32730	-2.30621	-0.12329
H	4.26758	-1.88061	0.76507
H	5.24469	-2.58138	-0.23601
O	0.45682	-0.22929	-2.53062
H	-0.19881	-0.88267	-2.23998
H	0.29115	-0.07230	-3.46950
O	2.72711	1.10509	0.69514
H	3.01078	0.73918	-0.18627
H	2.82156	2.07525	0.65883
O	3.26643	-0.22075	-1.57591
H	3.65577	-1.05127	-1.21819
H	2.41492	-0.44362	-1.98498
O	0.10928	1.49511	-0.19929
H	0.91870	1.18675	0.26757
H	0.22156	1.16170	-1.10737
O	4.01107	-0.82005	2.16180
H	3.47704	-1.12508	2.90451
H	3.55683	-0.02722	1.79100
O	1.81746	3.73951	0.18545
H	1.58312	4.55569	0.64168
H	0.98655	3.26239	-0.00417

Trichloromethane - 8 Water molecules

C	1.75740	-0.01067	-0.00777
H	0.73374	-0.39407	0.01681
Cl	1.76511	1.54456	-0.89159
Cl	2.29460	0.22294	1.69245
Cl	2.78086	-1.22740	-0.83554

O	-1.06592	-1.22352	0.40457
H	-1.33393	-1.98305	-0.16809
H	-0.94324	-1.57015	1.31006
O	-2.10674	-3.13101	-1.24666
H	-1.67117	-3.40703	-2.06159
H	-2.94218	-2.67334	-1.51101
O	5.66668	1.16336	-0.97236
H	5.15617	0.34367	-1.00624
H	5.00404	1.86424	-0.92689
O	-3.21049	0.40231	-0.16062
H	-2.42690	-0.10031	0.16886
H	-3.66103	0.80504	0.61037
O	-0.29474	-1.90696	3.02806
H	-0.22057	-2.78181	3.42897
H	0.57229	-1.48700	3.11642
O	-3.13661	3.11435	-0.85023
H	-2.95340	2.15238	-0.88961
H	-2.30686	3.56855	-1.03702
O	-4.25401	-1.55153	-1.76728
H	-5.16686	-1.78954	-1.56856
H	-4.02496	-0.77618	-1.19965
O	-4.28398	2.33258	1.53760
H	-5.18812	2.59114	1.74963
H	-3.97452	2.92314	0.81919

Trichloromethane-Water cluster models prepared by cutting out solvent molecules from water box optimized at B3LYP/6-311+G(2df,2p) theory level.

Trichloromethane - 5 Water molecules

C	-1.67118	-0.27703	-0.06337
H	-0.73450	-0.55019	-0.54733
Cl	-2.73833	-1.68760	0.00089
Cl	-1.26251	0.27491	1.60369
Cl	-2.41353	1.05591	-0.98675
O	2.80420	1.53306	-0.80070
H	2.11997	2.07016	-0.35016
H	3.21823	2.09660	-1.45935
O	0.74523	2.81513	0.54366
H	0.12610	3.34719	0.03472
H	0.20556	2.11883	0.94408
O	4.43539	-0.31065	0.58308
H	3.91308	0.40447	0.17597

H	4.70843	0.00866	1.44599
O	1.06002	-0.83153	-1.32027
H	1.61630	-1.54454	-0.95748
H	1.64120	-0.05942	-1.34160
O	2.95374	-2.59428	-0.12093
H	2.72515	-3.15707	0.62231
H	3.54321	-1.90115	0.23122

Trichloromethane - 7 Water molecules

C	-1.83277	-0.08257	-0.08128
H	-0.96974	-0.26143	-0.72463
Cl	-3.06793	-1.30964	-0.39835
Cl	-1.27807	-0.15387	1.62018
Cl	-2.46583	1.55745	-0.45370
O	1.26182	-3.31409	-1.34080
H	1.33583	-3.27501	-0.36386
H	0.77152	-4.11215	-1.54907
O	0.07926	3.56168	0.60430
H	-0.60753	2.97708	0.25583
H	-0.01174	3.52442	1.56071
O	3.14242	0.65257	-0.97906
H	3.00894	1.61544	-0.82796
H	3.80367	0.56689	-1.67104
O	2.64964	3.27199	-0.39635
H	2.80100	3.99873	-1.00538
H	1.73967	3.38695	-0.05599
O	3.56546	-0.99889	1.24997
H	3.46124	-0.37533	0.50608
H	3.99637	-0.51680	1.95875
O	0.60076	-0.61952	-1.81119
H	0.79151	-1.57542	-1.77094
H	1.43467	-0.18459	-1.58071
O	1.48725	-2.84408	1.37877
H	0.71470	-2.33382	1.63916
H	2.23455	-2.21404	1.41326

Trichloromethane - 10 Water molecules

C	-3.12664	0.24138	0.09637
H	-2.14026	0.68952	0.06139

Cl	-4.34067	1.53535	-0.00519
Cl	-3.26688	-0.87005	-1.29922
Cl	-3.26856	-0.65545	1.62926
O	-0.06372	1.10954	-0.00749
H	0.34934	1.99531	-0.07828
H	0.32534	0.69131	0.78691
O	0.72028	-0.33228	-2.14565
H	0.40371	0.23223	-1.39339
H	0.29140	-0.00040	-2.93925
O	0.07628	-2.88525	-1.11756
H	-0.87298	-2.93301	-0.97393
H	0.23154	-2.04107	-1.58105
O	3.90022	0.59481	1.77327
H	2.98549	0.41900	2.05891
H	4.19473	-0.24208	1.37329
O	1.17571	-0.19811	2.14652
H	0.72651	-0.28220	2.99176
H	1.32606	-1.12754	1.80604
O	3.87303	2.29580	-0.27586
H	3.91918	1.71895	0.53568
H	4.68964	2.80103	-0.30675
O	1.71792	-2.56798	1.12629
H	1.13634	-2.77807	0.36853
H	2.61402	-2.46866	0.76136
O	3.56311	-0.10427	-1.89799
H	3.68352	0.78541	-1.52701
H	2.61079	-0.18535	-2.08589
O	1.34683	3.45730	-0.36596
H	1.27295	4.22904	0.20022
H	2.28032	3.16690	-0.32500
O	4.26144	-1.72050	0.13087
H	5.01254	-2.28007	-0.08128
H	4.04473	-1.19846	-0.68448

Trichloromethane - 12 Water molecules

C	2.64758	-1.65879	0.20522
H	1.59990	-1.57907	-0.06633
Cl	3.62162	-0.92434	-1.10156
Cl	2.89182	-0.78206	1.74147
Cl	3.04719	-3.38081	0.39132
O	-2.80470	-2.75008	-1.29193
H	-2.66927	-3.06396	-2.19082
H	-3.45711	-2.02880	-1.34856
O	-0.40808	-1.24551	-0.46478
H	-1.10737	-1.87826	-0.68638
H	-0.47635	-0.53584	-1.15226
O	1.80902	2.07057	-2.39178
H	2.49413	1.52669	-1.99261
H	1.79268	2.90660	-1.88593
O	-4.17100	1.17820	0.99177

H	-4.53439	0.45217	0.46091
H	-3.41168	0.79093	1.45205
O	-5.04896	-1.11568	-0.61707
H	-5.84950	-0.97114	-1.12917
H	-5.20482	-1.91162	-0.06840
O	-2.17946	2.54954	-0.43498
H	-3.04777	2.34273	-0.04120
H	-1.82127	3.34402	-0.00830
O	-4.80137	-3.58208	0.57193
H	-4.58981	-3.77872	1.48822
H	-3.97256	-3.65552	0.07164
O	-1.28626	0.60677	1.42049
H	-1.47769	1.25170	0.71184
H	-0.94367	-0.17011	0.93171
O	-0.43253	4.37806	0.94685
H	-0.65249	5.15259	1.47142
H	-0.08510	3.70135	1.58300
O	-0.68067	0.81082	-2.20609
H	0.18008	1.25271	-2.35254
H	-1.23711	1.47065	-1.76289
O	1.63267	4.48249	-0.98151
H	1.54076	5.24778	-1.55421
H	0.90029	4.53023	-0.33800
O	0.42057	2.40148	2.56788
H	1.33867	2.12877	2.48861
H	-0.11845	1.63282	2.26994

Trichloromethane - 14 Water molecules

C	3.76093	-0.13182	0.05776
H	3.16667	-0.05665	0.96419
Cl	4.24328	-1.82763	-0.16390
Cl	2.71150	0.39927	-1.31446
Cl	5.16645	0.93383	0.19706
O	2.11118	-0.11398	2.68886
H	2.61469	-0.01579	3.50142
H	1.24767	0.33430	2.85741
O	-0.10376	-2.14107	2.80190
H	-0.32552	-2.57844	1.96563
H	0.80568	-1.82662	2.70691
O	0.80747	3.66469	-1.32981
H	1.55589	3.09159	-1.14409
H	0.21205	3.15707	-1.90914
O	-4.92282	1.42059	1.74889
H	-4.02231	1.74660	1.95459
H	-5.37882	2.16124	1.34128
O	-0.41185	0.61487	3.27772
H	-0.68883	-0.30792	3.14444
H	-1.08125	1.18851	2.86923
O	-5.28997	-0.93787	0.37990
H	-5.12552	-0.11814	0.89429

H	-6.17046	-1.23181	0.62773
O	-2.37877	2.44181	2.26807
H	-2.41761	3.11206	2.95654
H	-2.08695	2.91020	1.43995
O	-1.68003	3.69372	0.02923
H	-1.92455	3.24343	-0.79681
H	-0.73301	3.86718	-0.10779
O	-4.17022	-1.35743	-2.30986
H	-3.91823	-2.17955	-1.84800
H	-4.69045	-0.90644	-1.62909
O	-0.76316	-3.43068	0.27397
H	-0.49212	-4.35300	0.26024
H	-0.34122	-3.00486	-0.49373
O	-3.62983	-3.24301	-0.28996
H	-2.70979	-3.28773	0.00928
H	-4.05759	-2.54657	0.23113
O	0.37295	-1.98807	-1.91059
H	0.64313	-2.43737	-2.71796
H	1.11410	-1.41546	-1.66617
O	-1.79889	-0.14946	-2.91186
H	-1.14138	-0.73880	-2.52125
H	-2.67815	-0.54932	-2.71031
O	-1.46838	2.47094	-2.55509
H	-1.79096	2.88557	-3.35946
H	-1.60302	1.49418	-2.67591

Triethylamine-Water cluster models prepared manually optimized with PCM calculations at the B3LYP/6-31+G(d,p) theory level.

Triethylamine - 1 Water

N	-0.20436	-0.04349	0.03451
C	-1.46203	-0.76359	0.34668
H	-1.40081	-1.74165	-0.14369
H	-1.46685	-0.95427	1.42672
C	-0.11707	1.24129	0.76045
H	-0.40240	1.04013	1.79958
H	-0.84561	1.96759	0.36326
C	-2.77791	-0.07560	-0.04830
H	-3.61867	-0.73470	0.19414
H	-2.92777	0.86430	0.49166
H	-2.82162	0.13671	-1.12177
C	1.27339	1.87728	0.74035
H	1.26873	2.77668	1.36526
H	2.03107	1.19131	1.13213
H	1.57112	2.18115	-0.26778
C	0.02964	0.08887	-1.42730
H	-0.71065	-0.52199	-1.95391

H	-0.14866	1.12628	-1.74618
C	1.42569	-0.36091	-1.86970
H	1.53052	-0.23611	-2.95365
H	2.21559	0.21860	-1.38430
H	1.58669	-1.41747	-1.63248
O	1.62879	-1.81897	1.27231
H	1.29473	-1.91085	2.17392
H	1.01449	-1.17355	0.82395

Triethylamine - 4 Water molecules

N	0.23495	-0.08630	0.15559
C	1.10026	1.07354	-0.16618
H	0.55428	1.97392	0.13723
H	1.98298	1.00913	0.48150
C	0.92837	-1.36913	-0.08679
H	1.92818	-1.27939	0.35354
H	1.06848	-1.54284	-1.16672
C	1.55718	1.22068	-1.62576
H	2.14546	2.13875	-1.73091
H	2.18777	0.38509	-1.94445
H	0.70845	1.28979	-2.31438
C	0.22380	-2.58296	0.52032
H	0.84009	-3.47472	0.36436
H	0.06910	-2.45646	1.59651
H	-0.74720	-2.77026	0.05186
C	-1.09663	-0.00434	-0.49971
H	-1.19588	0.98222	-0.96382
H	-1.15927	-0.73445	-1.31977
C	-2.26939	-0.20212	0.46580
H	-3.21717	-0.13437	-0.08069
H	-2.23211	-1.17680	0.95986
H	-2.26871	0.57072	1.24121
O	0.09216	0.24051	2.97008
H	1.01530	0.32092	3.24266
H	0.11617	0.09858	1.98305
O	3.11281	6.24734	-0.17442
H	3.41104	6.92529	0.44685
H	2.93509	6.72396	-0.99653
O	-9.10978	-0.32230	-0.77084
H	-9.79578	0.31224	-1.01856
H	-9.58782	-1.13398	-0.55350
O	5.34954	-4.46010	-0.76334
H	5.06381	-5.38362	-0.77318
H	6.31365	-4.49729	-0.82304

Triethylamine - 4 Water molecules preoptimized with PM3

N	-0.20319	0.13579	0.58529
C	0.21124	-0.72473	1.71973
H	0.46130	-1.71033	1.31585
H	1.14741	-0.30409	2.10395
C	-0.34498	1.54974	1.00169
H	0.50098	1.77378	1.66057
H	-1.26063	1.69345	1.59922
C	-0.78443	-0.88788	2.87839
H	-0.34030	-1.53182	3.64509
H	-1.02789	0.06931	3.34999
H	-1.71969	-1.35658	2.55594
C	-0.34076	2.54819	-0.15890
H	-0.34363	3.56691	0.24306
H	0.55279	2.43075	-0.78004
H	-1.22048	2.45017	-0.80154
C	-1.41371	-0.34731	-0.11545
H	-2.29728	-0.28534	0.54251
H	-1.59833	0.33844	-0.94634
C	-1.28781	-1.76388	-0.67825
H	-2.16421	-1.98460	-1.29627
H	-0.39700	-1.86221	-1.30726
H	-1.24047	-2.52562	0.10565
O	2.01018	-0.13193	-1.13064
H	1.22069	-0.00313	-0.53032
H	2.64667	0.57956	-0.92207
O	3.14263	-2.62114	-1.20956
H	3.38851	-2.86861	-0.30927
H	2.73325	-1.72410	-1.13690
O	-5.52866	0.35618	-1.57831
H	-4.79508	-0.27358	-1.58563
H	-6.19689	-0.03224	-2.15894
O	3.92028	1.91135	-0.54200
H	3.94623	2.64915	-1.16712
H	4.83369	1.59976	-0.47632

Triethylamine - 5 Water molecules

N	1.46942	-0.33866	0.37728
C	1.98411	0.93806	-0.17277
H	1.32631	1.73337	0.19554
H	2.96868	1.11168	0.27802
C	2.36385	-1.47129	0.05724
H	3.38635	-1.14148	0.27472
H	2.32655	-1.71047	-1.01858
C	2.10527	1.04233	-1.70093
H	2.45056	2.04662	-1.96956
H	2.82615	0.32520	-2.10523
H	1.14400	0.87779	-2.19915
C	2.07033	-2.74236	0.85513
H	2.82386	-3.50078	0.61770
H	2.10223	-2.55218	1.93260
H	1.09166	-3.16496	0.60812

C	0.05060	-0.58725	0.01095
H	-0.34848	0.31400	-0.46547
H	-0.01023	-1.38384	-0.74487
C	-0.84130	-0.93351	1.20738
H	-1.86883	-1.10759	0.86763
H	-0.49906	-1.83323	1.72593
H	-0.85759	-0.11159	1.93040
O	1.80906	0.20686	3.14016
H	2.72854	0.49423	3.21028
H	1.67125	-0.01646	2.17778
O	3.66555	5.90940	0.36863
H	3.49705	6.37315	1.19998
H	4.04005	6.57974	-0.21870
O	-12.06710	-1.03703	0.79888
H	-12.57306	-1.74405	0.37611
H	-12.52124	-0.87205	1.63621
O	6.80983	-3.00980	-2.06274
H	6.99636	-3.82987	-2.53945
H	7.60515	-2.47150	-2.17336
O	-7.80616	1.41659	-2.66796
H	-8.33025	0.62042	-2.50670
H	-7.94836	1.97045	-1.88849

Triethylamine - 5 Water molecules preoptimized with PM3

N	-1.41718	-0.67369	-0.00427
C	-1.76623	-2.10055	0.19835
H	-2.19370	-2.19938	1.20066
H	-2.57636	-2.32602	-0.50436
C	-1.04181	-0.40125	-1.41047
H	-1.73898	-0.96346	-2.04113
H	-0.03231	-0.78743	-1.62962
C	-0.64052	-3.13098	0.02134
H	-1.04535	-4.13595	0.18224
H	-0.21201	-3.10354	-0.98544
H	0.17091	-2.98363	0.74125
C	-1.11155	1.07742	-1.80091
H	-0.91400	1.17654	-2.87358
H	-2.10338	1.49304	-1.59712
H	-0.36952	1.68647	-1.27629
C	-0.39062	-0.17393	0.93720
H	0.57932	-0.66767	0.75588
H	-0.24309	0.88669	0.71755
C	-0.77244	-0.31333	2.41175
H	-0.02889	0.20451	3.02624
H	-1.75024	0.13699	2.61171
H	-0.80062	-1.35605	2.74188
O	-3.86099	0.58576	0.58800
H	-2.98821	0.15816	0.35171
H	-4.48897	0.36418	-0.12711
O	-3.76854	3.24340	1.22071

H	-3.78193	2.29112	0.95436
H	-3.35419	3.71295	0.48582
O	8.88982	1.60969	-0.63488
H	7.96991	1.65008	-0.33846
H	8.85698	1.73493	-1.59351
O	-5.73360	-0.02933	-1.48143
H	-6.41615	-0.67238	-1.24398
H	-6.21276	0.73365	-1.83366
O	10.34914	-0.64662	0.23758
H	11.07442	-0.30881	0.77851
H	9.85169	0.14338	-0.05705

Triethylamine-Water cluster models prepared by cutting out solvent molecules from water box optimized with PCM calculations at the B3LYP/6-31+G(d,p) theory level.

Triethylamine - 1 Water

N	0.89661	0.42171	-0.33287
C	2.01488	0.11690	0.57765
H	2.72124	-0.52689	0.04326
H	2.54197	1.06105	0.75965
C	-0.00048	1.45816	0.20168
H	0.61332	2.15718	0.78068
H	-0.73872	1.03349	0.90775
C	1.66689	-0.53124	1.93036
H	2.58362	-0.67532	2.51317
H	0.99235	0.09748	2.52131
H	1.19612	-1.51232	1.80853
C	-0.73643	2.24692	-0.88561
H	-1.35132	3.02945	-0.42687
H	-0.02280	2.72422	-1.56557
H	-1.40277	1.61466	-1.48086
C	0.15675	-0.76153	-0.79560
H	-0.31723	-1.29933	0.04788
H	-0.66278	-0.40554	-1.42669
C	0.99935	-1.74238	-1.61361
H	0.35458	-2.52607	-2.02558
H	1.49169	-1.23054	-2.44785
H	1.76977	-2.23550	-1.01280
O	-4.13248	-0.97988	0.63330
H	-3.18753	-0.82546	0.49832
H	-4.40367	-0.31449	1.28017

Triethylamine - 3 Water molecules

N	-1.31414	-0.48272	-0.77135
C	-1.77113	-1.70369	-1.45881
H	-2.77036	-1.51121	-1.86322
H	-1.11445	-1.84428	-2.32561
C	0.10392	-0.54959	-0.38387
H	0.62620	-1.13750	-1.14685
H	0.23953	-1.08887	0.57247
C	-1.80245	-3.00601	-0.63790
H	-2.13169	-3.83311	-1.27691
H	-0.81310	-3.26286	-0.24458
H	-2.49727	-2.94468	0.20619
C	0.77464	0.82412	-0.28838
H	1.83361	0.70486	-0.03295
H	0.70920	1.35345	-1.24496
H	0.32176	1.45622	0.48198
C	-2.17097	-0.06591	0.34894
H	-2.19807	-0.83242	1.14646
H	-1.71099	0.81974	0.79714
C	-3.60103	0.29221	-0.06064
H	-4.13630	0.70790	0.79988
H	-3.60181	1.04250	-0.85912
H	-4.16799	-0.57679	-0.40872
O	8.05125	1.99887	-1.10221
H	7.25466	2.42009	-0.75202
H	7.88868	1.04959	-1.01820
O	3.40729	-2.54510	2.56755
H	3.21021	-3.23102	3.21967
H	2.57183	-2.39276	2.10540
O	-3.02757	4.67211	1.08365
H	-3.03182	3.74365	0.81415
H	-3.92860	4.98262	0.92135

Triethylamine - 4 Water molecules

N	0.28472	-1.11192	-0.06779
C	1.00254	-2.39045	-0.21694
H	2.01046	-2.26301	0.19160
H	0.49969	-3.11454	0.43515
C	-1.14901	-1.22922	-0.37819
H	-1.47064	-2.23173	-0.07476
H	-1.33799	-1.15691	-1.46547
C	1.10488	-2.98068	-1.63548
H	1.63332	-3.93984	-1.59532
H	0.11722	-3.16284	-2.07234

H	1.66051	-2.32621	-2.31517
C	-2.01562	-0.20083	0.35488
H	-3.07206	-0.36308	0.11278
H	-1.89215	-0.29491	1.43915
H	-1.76927	0.82788	0.07300
C	0.91010	0.01572	-0.77489
H	0.94170	-0.15703	-1.86727
H	0.26779	0.88868	-0.62579
C	2.31354	0.36264	-0.27386
H	2.66155	1.27992	-0.76100
H	2.31023	0.52988	0.80893
H	3.04394	-0.42156	-0.49596
O	-5.49568	-1.50679	2.60334
H	-4.64917	-1.06568	2.75701
H	-5.60524	-1.50449	1.64273
O	-5.08221	2.49904	-1.55635
H	-5.16650	3.46182	-1.53385
H	-4.27378	2.30763	-1.06185
O	7.40364	0.55272	1.14543
H	6.74589	-0.09853	1.42467
H	8.24337	0.22438	1.49410
O	0.83946	4.69380	0.39894
H	1.13515	3.77416	0.35964
H	1.65270	5.21610	0.42052

Triethylamine - 7 Water molecules

N	0.70918	0.30480	1.14380
C	-0.01543	0.24478	2.43448
H	-0.99663	0.70714	2.28864
H	-0.20318	-0.81564	2.63820
C	1.95457	-0.49304	1.18010
H	1.73079	-1.41093	1.73443
H	2.74462	0.03322	1.74235
C	0.66867	0.88089	3.65479
H	0.03203	0.74567	4.53586
H	1.63661	0.41763	3.87070
H	0.82737	1.95623	3.52466
C	2.48888	-0.87556	-0.20281
H	3.35849	-1.53115	-0.08574
H	1.73139	-1.41277	-0.78220
H	2.81283	-0.00634	-0.78307
C	0.96208	1.68134	0.66723
H	1.66160	2.20740	1.33986
H	1.46703	1.59857	-0.29892
C	-0.30188	2.52196	0.48042
H	-0.03796	3.46755	-0.00446
H	-1.02817	2.00544	-0.15570
H	-0.78872	2.76638	1.42934
O	2.95232	-5.15404	-0.51148
H	2.34746	-4.47696	-0.84383

H	3.76939	-4.67846	-0.30915
O	6.50481	-0.97613	-2.16706
H	6.94078	-0.45738	-2.85653
H	5.65517	-0.53740	-2.02425
O	-7.88796	-0.59719	-1.78829
H	-7.00272	-0.22421	-1.58394
H	-8.52377	0.06833	-1.49615
O	-5.35873	0.42980	-1.21084
H	-4.80439	-0.12907	-0.62612
H	-4.81358	0.63191	-1.98274
O	3.28721	4.53760	-2.19092
H	2.55110	4.21694	-1.65236
H	2.92302	5.27696	-2.69624
O	-3.80866	-1.16706	0.51347
H	-4.10106	-2.09022	0.52632
H	-3.85942	-0.86784	1.43316
O	-1.07868	-1.04227	-0.63770
H	-1.95042	-1.04701	-0.20711
H	-0.46783	-0.55539	-0.02423
